# Supplementary material for: Severity of acute SARS-CoV-2 infection and risk of new-onset autoimmune disease: A RECOVER initiative study in nationwide U.S. cohorts
Source: PLoS One. 2025 Jun 4;20(6):e0324513. doi: 10.1371/journal.pone.0324513 (PMC12136303; doi:10.1371/journal.pone.0324513)
Supplement: S1 Appendix — (PDF) [file pone.0324513.s001.pdf]

RECOVER

Researching COVID to Enhance Recovery

PCORnet RECOVER EHR

Cohort Inclusion Criteria

March 2022

COHORT INCLUSION CRITERIA

The cohort inclusion criteria defining the data to be submitted to the RECOVER CC is as follows:

- Anyone with a COVID test (Ag, Ab), regardless of result
  - LOINC codes for COVID tests: 95942-9, 97099-6, 95941-1, 95380-2, 95423-0, 95422-2, 98733-9, 99771-8, 98846-9, 98847-7, 99774-2, 99773-4, 95209-3, 94763-0, 94661-6, 95825-6, 98069-8, 94762-2, 95542-7, 94769-7, 96118-5, 94504-8, 94503-0, 94558-4, 96119-3, 97097-0, 96094-8, 98080-5, 96896-6, 96764-6, 96763-8, 94562-6, 94768-9, 95427-1, 94720-0, 95125-1, 96742-2, 94761-4, 94563-4, 94507-1, 95429-7, 94505-5, 94547-7, 95416-4, 94564-2, 94508-9, 95428-9, 94506-3, 96895-8, 100157-7, 96957-6, 95521-1, 96898-2, 94510-5, 94311-8, 94312-6, 95522-9, 94760-6, 96986-5, 95409-9, 94533-7, 94756-4, 94757-2, 95425-5, 96448-6, 96958-4, 94766-3, 94316-7, 94307-6, 94308-4, 99596-9, 95411-5, 95410-7, 97098-8, 98132-4, 98494-8, 96899-0, 94644-2, 94511-3, 94559-2, 95824-9, 94639-2, 97104-4, 98131-6, 98493-0, 94646-7, 94645-9, 96120-1, 94534-5, 96091-4, 94314-2, 96123-5, 99314-7, 94745-7, 94746-5, 94819-0, 94565-9, 94759-8, 95406-5, 96797-6, 95608-6, 94500-6, 95424-8, 94845-5, 94822-4, 94660-8, 94309-2, 96829-7, 96897-4, 94531-1, 95826-4, 94306-8, 96900-6, 94642-6, 94643-4, 94640-0, 95609-4, 96765-3, 94767-1, 94641-8, 96752-1, 96751-3, 99597-7, 96603-6, 98732-1, 98734-7, 96894-1, 95970-0, 100156-9, 96741-4, 96755-4, 94764-8, 99772-6, 95971-8, 95974-2, 95972-6, 95973-4, 94509-7, 96121-9, 94758-0, 95823-1, 94765-5, 94315-9, 96122-7, 94313-4, 94310-0, 94502-2, 94647-5, 94532-9
  - Please add any local variation in coding that identifies SARS-CoV-2/COVID19 RT-PCR or direct viral antigen testing.

- Anyone with a COVID-19 specific diagnosis.
  - ICD-10-CM codes for COVID diagnoses: B97.29, U07.1, B34.2, B97.2, B97.21, J12.81, U04, U04.9, U07.2, U07.2

- Anyone with a COVID-19 vaccine.
  - CPT codes for COVID vaccines: 91300, 0001A, 0002A, 59267100001, 2468235, 2468232, 2468230, 208, 0003A, 0004A, 59267100002, 59267100003, 91305, 0051A, 0052A, 0053A, 0054A, 91307, 0071A, 0072A, 91301, 0011A, 0012A, 80777027310, 2470234, 2470233, 2470232, 207, 0013A, 80777027315, 80777027398, 80777027399, 91306, 0064A, 212, 91303, 0031A, 59676058005, 2479835, 59676058015, 0034A)
  - CVX codes for COVID19 vaccines: 207, 208, 210, 212, 213

- RxNorm CUIs for COVID-19 vaccines: 2468230, 2468231, 2468232, 2468233, 2468234, 2468235, 2470232, 2470233, 2470234, 2479830, 2479831, 2479832, 2479833, 2479834, 2479835, 2583742, 2583743

- Anyone with a set of respiratory diagnoses after 01/01/2019.
  - Respiratory diagnoses codes: 480.0, 480.1, 480.8, 480.9, 486, 487.0, 480.0, J12.0, J12.1, J12.89, J12.9, J12, J12.8, J18.8, P23.0, A01.03, A02.22, A37.01, A37.11, A37.81, A37.91, A54.84, B01.2, B05.2, B06.81, B20.6, B77.81, J12, J12.2, J12.3, J12.8, J12.81, J13, J14, J14, J15, J15.0, J15.1, J15.2, J15.20, J15.21, J15.211, J15.212, J15.29, J15.3, J15.4, J15.5, J15.6, J15.7, J15.8, J15.8, J15.9, J15.9, J16, J16.0, J16.8, J17, J17.0, J17.1, J17.2, J17.3, J17.8, J18, J18, J18.0, J18.0, J18.1, J18.1, J18.2, J18.2, J18.9, J18.9, J84.11, J84.111, J84.116, J84.117, J84.2, J85.1, J95.851, J41, J40, J41.0, J41.1, J41.8, J42, J68.0, 491, 491.1, 491.8, 491.9, 506, 490, J98.8, J84.115, J22, J44.0, J47.0, J20.2, J20.1, J20.0, J20.7, J20.3, J20.6, J20.5, J20.4, J20.8, J20.9, J21.0, J21.1, J21.8, J21.9, J20, J21, 041, 041.5, 041.81, 079.1, 079.2, 079.3, 079.6, 079.89, 466, 466.11, 466.19, 519.8, J09, J09, J09.01, J09.010, J09.018, J09.019, J09.02, J09.03, J09.090, J09.091, J09.092, J09.098, J09.11, J09.110, J09.118, J09.119, J09.12, J09.13, J09.190, J09.191, J09.192, J09.198, J09.X, J09.X1, J09.X2, J09.X3, J09.X9, J10, J10, J10.0, J10.0, J10.00, J10.01, J10.08, J10.1, J10.1, J10.2, J10.8, J10.8, J10.81, J10.82, J10.83, J10.89, J11, J11, J11.0, J11.0, J11.00, J11.08, J11.1, J11.1, J11.2, J11.8, J11.8, J11.81, J11.82, J11.83, J11.89, 518.82, J80, R06.03, P22, P22.0, P22.8, P22.9, P28.11, R06.03, J95.82, J95.821, J95.822, J96, J96.0, J96.00, J96.01, J96.02, J96.1, J96.10, J96.11, J96.12, J96.2, J96.20, J96.21, J96.22, J96.9, J96.90, J96.91, J96.92, P28.5, P28.81, R09.2, 786.2, R05, B33.8, B34.8, B97.89, J00, J02.9, J04.0, J04.1, J04.10, J04.11, J04.2, J04.3, J04.30, J04.31, J05.0, J05.0, J05.1, J05.10, J05.11, J06.0, J06.9, J39.8, J39.9, R05, J01.0, J01.00, J01.01, J01.1, J01.10, J01.11, J01.2, J01.20, J02.21, J01.3, J01.30, J01.31, J01.4, J01.40, J01.41, J01.8, J01.80, J01.81, J01.9, J01.90, J01.91, J02, J02.0, J02.8, J03, J03.0, J03.00, J03.01, J03.8, J03.80, J03.81, J03.9, J03.90, J03.91, 786.05, R06.02, 780.60, 780.61, R50.9, R50.81, R50, R50.8, R50.2, R50.84, R50.9, R56.00

- Anyone with a PASC diagnosis code (U09.9).

- Anyone with the following ICD-10 codes for MIS-C or MIS-A, Kawasaki disease, myocarditis, or pericarditis:
  - MIS-C or MIS-A: M35.81
  - Kawasaki disease: M30.3
  - Myocarditis: B33.22, I40, I40.0, I40.1, I40.8, I40.9, I51.4

- Pericarditis: B33.23, I30, I30.0, I30.1, I30.8, I30.9, I31.9

For the patient set defined by the above criteria, sites will need to submit records for included patients from all tables in the data model, with a few limitations, as shown below.

PCORnet	OMOP
Records from these tables can be limited to after 1/1/2017 if desired:	Records from these tables can be limited to after 1/1/2017 if desired:
Labs	Measurement
Vitals	
Obs_gen	
Obs_clin	

Please be sure to submit all necessary records from tables like Provider, Location, and LDS_Address_History.

Please note that if your site is participating in the COVID CDC project, your site can share the same data submissions for the first RECOVER data submission. We are also happy to accept your full PCORnet CDM for the first submission given the expedited timeframes.
