# Supplementary material for: Severity of acute SARS-CoV-2 infection and risk of new-onset autoimmune disease: A RECOVER initiative study in nationwide U.S. cohorts
Source: PLoS One. 2025 Jun 4;20(6):e0324513. doi: 10.1371/journal.pone.0324513 (PMC12136303; doi:10.1371/journal.pone.0324513)
Supplement: S1 Table — Table from Rao et al. eClinicalMedicine. (2025) 80 in Press [1]; Forrest et al. Pediatrics (2022) 149 (4): e2021055765 [2]. (DOCX) [file pone.0324513.s010.docx]

| **Mild** | **Moderate** | **Severe** |
| --- | --- | --- |
| Abdominal pain | Acute bronchitis | Acute respiratory distress syndrome |
| Anorexia | Bronchiolitis | Acute kidney injury |
| Cough | Dehydration | Acute liver failure |
| Diarrhea | Gastroenteritis | Death |
| Fever/chills | Pneumonia | Encephalopathy/encephalitis |
| Headache | Use of IV fluids during the first 6 hours of an emergency department visit | Myocarditis |
| Loss of taste or smell |  | Pericarditis |
| Fatigue |  | Respiratory failure |
| Myalgia |  | Sepsis |
| Nasal Congestion |  | Shock |
| Nausea |  | Thromboembolism |
| Sore throat |  | ICU admission |
| Respiratory symptoms |  | Mechanical ventilation |
| Vomiting |  | Vasopressors and inotropes |
|  |  | Respiratory failure |
|  |  | Sepsis |
|  |  | Shock |

## **References:**

1. Rao, Suchitra,Diaz, Ivan.Snowden, al. Je. eClinicalMedicine. 2025;80:103042.

2. Razzaghi H, Forrest C, al KHe. Vaccine Effectiveness Against Long COVID in Children. Pediatrics. 2024;153(4).
