# Supplementary material for: Severity of acute SARS-CoV-2 infection and risk of new-onset autoimmune disease: A RECOVER initiative study in nationwide U.S. cohorts
Source: PLoS One. 2025 Jun 4;20(6):e0324513. doi: 10.1371/journal.pone.0324513 (PMC12136303; doi:10.1371/journal.pone.0324513)
Supplement: S3 Table — (DOCX) [file pone.0324513.s012.docx]

|  | **aHR** | **95% CI** | **p-value** |
| --- | --- | --- | --- |
| **Model 1:** Age, sex, and race/ethnicity | | | |
|  |  |  |  |
| Outpatient | Reference | -- | -- |
| Emergency department | 0.91 | 0.84 - 0.99 | 0.028 |
| Hospitalized | 0.91 | 0.83 - 0.99 | 0.037 |
| Hospitalized and on ventilator | 1.29 | 1.17 - 1.40 | <0.001 |
|  |  |  |  |
| 21-35 | Reference | -- | -- |
| 36-45 | 1.38 | 1.29 - 1.46 | 0 |
| 46-65 | 1.59 | 1.51 - 1.67 | 0 |
| 66+ | 1.82 | 1.75 - 1.89 | 0 |
| **Male Sex** | 0.54 | 0.49 - 0.60 | 0 |
|  |  |  |  |
| Non-Hispanic Asian | 0.76 | 0.60 - 0.93 | 0.001 |
| Non-Hispanic Black | 0.91 | 0.84 - 0.98 | 0.011 |
| Non-Hispanic White | Reference | -- | -- |
| Non-Hispanic Other/Multiple | 0.93 | 0.83 - 1.03 | 0.167 |
| Hispanic or Latino | 0.89 | 0.81 - 0.96 | 0.001 |
| Missing/unknown | 0.55 | 0.47 - 0.64 | 0 |
| **Model 2:** Full adjustment^8^ | | | |
|  |  |  |  |
| Outpatient | Reference | -- | -- |
| Emergency department | 0.95 | 0.88 - 1.03 | 0.237 |
| Hospitalized | 0.82 | 0.73 - 0.90 | <0.001 |
| Hospitalized and on ventilator | 1.14 | 1.02 - 1.26 | 0.032 |
|  |  |  |  |
| 21-35 | Reference | -- | -- |
| 36-45 | 1.22 | 1.14 - 1.30 | <0.001 |
| 46-65 | 1.27 | 1.18 - 1.35 | 0 |
| 66+ | 1.20 | 1.13 - 1.28 | <0.001 |
| **Male Sex** | 0.62 | 0.57 - 0.67 | 0 |
|  |  |  |  |
| Non-Hispanic Asian | 0.88 | 0.71 - 1.05 | 0.132 |
| Non-Hispanic Black | 0.85 | 0.78 - 0.92 | <0.001 |
| Non-Hispanic White | Reference | -- | -- |
| Non-Hispanic Other/Multiple | 1.00 | 0.89 - 1.11 | 0.999 |
| Hispanic or Latino | 0.95 | 0.87 - 1.02 | 0.148 |
| Missing/unknown | 0.77 | 0.68 - 0.86 | 0 |
|  |  |  |  |
| 0 | Reference | -- | -- |
| 1-3 | 1.40 | 1.34 - 1.46 | 0 |
| 4+ | 1.52 | 1.44- 1.61 | 0 |
|  |  |  |  |
| Never | Reference | -- | -- |
| Ever | 0.93 | 0.86 - 0.99 | 0.026 |
| Missing/Unknown | 0.98 | 0.91 - 1.04 | 0.452 |
| **Prior substance abuse** | 1.36 | 1.27 - 1.45 | 0 |
|  |  |  |  |
| underweight | 0.99 | 0.74 - 1.25 | 0.959 |
| normal weight | Reference | -- | -- |
| overweight | 1.02 | 0.94- 1.10 | 0.680 |
| moderately obese | 0.96 | 0.87 - 1.04 | 0.304 |
| severely obese | 0.95 | 0.85 - 1.05 | 0.363 |
| morbidly obese | 0.94 | 0.84 - 1.04 | 0.235 |
| missing/unknown | 0.80 | 0.71 - 0.90 | <0.001 |
| **Prior glucocorticoid use** ^5^ | 1.36 | 1.29- 1.42 | 0 |
|  |  |  |  |
| Private | Reference | -- | -- |
| Medicare/Medicaid | 0.97 | 0.89 - 1.05 | 0.463 |
| Other | 0.35 | 0.01 -0.71 | 0 |
| Missing/Unknown | 0.91 | 0.84 - 0.97 | 0.004 |
|  |  |  |  |
| Quartile 1 | Reference | -- | -- |
| Quartile 2 | 1.47 | 1.08 - 1.85 | 0.054 |
| Quartile 3 | 1.37 | 0.99 - 1.76 | 0.106 |
| Quartile 4 | 1.59 | 1.21 - 1.97 | 0.017 |
|  |  |  |  |
| 1-2 | Reference | -- | -- |
| 0 | 0.63 | 0.53 - 0.73 | 0 |
| 3-5 | 1.19 | 1.09 - 1.29 | <0.001 |
| 6-10 | 1.38 | 1.28 - 1.48 | 0 |
| 11-20 | 1.73 | 1.63 - 1.82 | 0 |
| 21+ | 2.27 | 2.17 - 2.37 | 0 |
|  |  |  |  |
| Apr-June 2020 | Reference | -- | -- |
| Jul-Sep 2020 | 0.95 | 0.87 - 1.03 | 0.241 |
| Oct-Dec 2020 | 0.90 | 0.83 - 0.97 | 0.003 |
| Jan -Apr 2021 | 0.93 | 0.86 - 1.00 | 0.073 |
| **Model 3:** Full adjustment^8^- sex-stratified (male) | | | |
| **COVID severity** |  |  |  |
| Outpatient | Reference | -- | -- |
| Emergency department | 0.93 | 0.79 - 1.08 | 0.351 |
| Hospitalized | 0.97 | 0.84 - 1.11 | 0.695 |
| Hospitalized and on ventilator | 1.30 | 1.11 - 1.49 | 0.006 |
| **Age** |  |  |  |
| 21-35 | Reference | -- | -- |
| 36-45 | 1.20 | 1.03 - 1.37 | 0.035 |
| 46-65 | 1.22 | 1.06 - 1.38 | 0.016 |
| 66+ | 1.28 | 1.14 - 1.43 | <0.001 |
| **Race/ethnicity**^2^ |  |  |  |
| Non-Hispanic Asian | 0.82 | 0.51 - 1.12 | 0.199 |
| Non-Hispanic Black | 0.85 | 0.71 - 0.99 | 0.023 |
| Non-Hispanic White | Reference | -- | -- |
| Non-Hispanic Other/Multiple | 1.07 | 0.88 - 1.26 | 0.493 |
| Hispanic or Latino | 0.90 | 0.76- 1.04 | 0.158 |
| Missing/unknown | 0.71 | 0.55 - 0.87 | <0.001 |
| **Charlson comorbidity index ^3^** |  |  |  |
| 0 | Reference | -- | -- |
| 1-3 | 1.47 | 1.35 - 1.59 | 0 |
| 4+ | 1.60 | 1.45 - 1.74 | 0 |
| **Smoking status** |  |  |  |
| Never | Reference | -- | -- |
| Ever | 0.96 | 0.85 - 1.08 | 0.531 |
| Missing/Unknown | 0.88 | 0.77 - 1.00 | 0.037 |
| **Prior substance abuse** | 1.37 | 1.23 - 1.51 | <0.001 |
| **Body mass index** ^4^ |  |  |  |
| underweight | 0.36 | 0.23 - 1.24 | 0.023 |
| normal weight | Reference | -- | -- |
| overweight | 1.00 | 0.85 - 1.15 | 0.983 |
| moderately obese | 0.93 | 0.77 - 1.09 | 0.354 |
| severely obese | 0.94 | 0.75 - 1.13 | 0.522 |
| morbidly obese | 0.81 | 0.58 - 1.03 | 0.062 |
| missing/unknown | 0.85 | 0.67 - 1.02 | 0.065 |
| **Prior glucocorticoid use** ^5^ | 1.22 | 1.10 - 1.35 | 0.002 |
| **Medical insurance** |  |  |  |
| Private | Reference | -- | -- |
| Medicare/Medicaid | 0.99 | 0.84 - 1.13 | 0.889 |
| Other | 0.53 | 0.06 - 1.01 | 0.010 |
| Missing/Unknown | 0.94 | 0.82 - 1.06 | 0.296 |
| **RECOVER site size**^7^ |  |  |  |
| Quartile 1 | Reference | -- | -- |
| Quartile 2 | 1.45 | 0.73 - 2.16 | 0.311 |
| Quartile 3 | 1.57 | 0.86 - 2.28 | 0.214 |
|  | 1.79 | 1.09 - 2.49 | 0.104 |
| **Prior encounters** ^6^ |  |  |  |
| 1-2 | Reference | -- | -- |
| 0 | 0.55 | 0.37 - 0.72 | 0 |
| 3-5 | 1.17 | 1.00 - 1.34 | 0.070 |
| 6-10 | 1.42 | 1.25 - 1.59 | <0.001 |
| 11-20 | 1.80 | 1.63 - 1.97 | 0 |
| 21+ | 2.29 | 2.12 - 2.47 | 0 |
| **Index date period** |  |  |  |
| Apr-June 2020 | Reference | -- | -- |
| Jul-Sep 2020 | 1.07 | 0.93 - 1.22 | 0.349 |
| Oct-Dec 2020 | 1.01 | 0.88 - 1.14 | 0.84 |
| Jan -Apr 2021 | 1.01 | 0.88 - 1.15 | 0.866 |
| **Model 4:** Full adjustment^8^- sex-stratified (female) | | | |
| **COVID severity** |  |  |  |
| Outpatient | Reference | -- | -- |
| Emergency department | 0.96 | 0.87 - 1.05 | 0.419 |
| Hospitalized | 0.73 | 0.62 - 0.84 | 0 |
| Hospitalized and on ventilator | 1.06 | 0.90 - 1.21 | 0.488 |
| **Age** |  |  |  |
| 21-35 | Reference | -- | -- |
| 36-45 | 1.23 | 1.13 - 1.32 | <0.001 |
| 46-65 | 1.29 | 1.19 - 1.38 | <0.001 |
| 66+ | 1.16 | 1.08 - 1.25 | <0.001 |
| **Race/ethnicity**^2^ |  |  |  |
| Non-Hispanic Asian | 0.91 | 0.71 - 1.11 | 0.339 |
| Non-Hispanic Black | 0.85 | 0.76 - 0.94 | <0.001 |
| Non-Hispanic White | Reference | -- | -- |
| Non-Hispanic Other/Multiple | 0.97 | 0.83 - 1.11 | 0.639 |
| Hispanic or Latino | 0.96 | 0.87- 1.05 | 0.422 |
| Missing/unknown | 0.81 | 0.69 - 0.92 | <0.001 |
| **Charlson comorbidity index ^3^** |  |  |  |
| 0 | Reference | -- | -- |
| 1-3 | 1.36 | 1.29 - 1.44 | 0 |
| 4+ | 1.48 | 1.38 - 1.58 | 0 |
| **Smoking status** |  |  |  |
| Never | Reference | -- | -- |
| Ever | 0.90 | 0.82 - 0.98 | 0.012 |
| Missing/Unknown | 1.02 | 0.95 - 1.10 | 0.567 |
| **Prior substance abuse** | 1.35 | 1.23 - 1.46 | <0.001 |
| **Body mass index** ^4^ |  |  |  |
| underweight | 1.18 | 0.92 - 1.45 | 0.212 |
| normal weight | Reference | -- | -- |
| overweight | 1.02 | 0.93 - 1.12 | 0.623 |
| moderately obese | 0.97 | 0.87 - 1.07 | 0.560 |
| severely obese | 0.97 | 0.85 - 1.08 | 0.560 |
| morbidly obese | 0.99 | 0.87 - 1.10 | 0.83 |
| missing/unknown | 0.78 | 0.67 - 0.90 | <0.001 |
| **Prior glucocorticoid use** ^5^ | 1.41 | 1.33 - 1.49 | 0 |
| **Medical insurance** |  |  |  |
| Private | Reference | -- | -- |
| Medicare/Medicaid | 0.96 | 0.87 - 1.06 | 0.444 |
| Other | 0.23 | 0.16 - 0.78 | <0.001 |
| Missing/Unknown | 0.89 | 0.81 - 0.97 | 0.005 |
| **RECOVER site size**^7^ |  |  |  |
| Quartile 1 | Reference | -- | -- |
| Quartile 2 | 1.49 | 1.02 - 1.95 | 0.093 |
| Quartile 3 | 1.31 | 0.85 - 1.77 | 0.253 |
| Quartile 4 | 1.52 | 1.07 - 1.98 | 0.069 |
| **Prior encounters** ^6^ |  |  |  |
| 1-2 | Reference | -- | -- |
| 0 | 0.68 | 0.55 - 0.81 | 0 |
| 3-5 | 1.19 | 1.08 - 1.31 | 0.003 |
| 6-10 | 1.36 | 1.24 - 1.47 | <0.001 |
| 11-20 | 1.70 | 1.58 - 1.81 | 0 |
| 21+ | 2.26 | 2.14 - 2.38 | 0 |
| **Index date period** |  |  |  |
| Apr-June 2020 | Reference | -- | -- |
| Jul-Sep 2020 | 0.91 | 0.81 - 1.00 | 0.045 |
| Oct-Dec 2020 | 0.85 | 0.77 - 0.94 | <0.001 |
| Jan -Apr 2021 | 0.90 | 0.82 - 0.99 | 0.025 |
| **Model 5:** Full adjustment^8^- no prior^9^ | | | |
| **COVID severity** |  |  |  |
| Outpatient | Reference | -- | -- |
| Emergency department | 0.90 | 0.81 - 0.99 | 0.017 |
| Hospitalized | 0.82 | 0.72 - 0.92 | <0.001 |
| Hospitalized and on ventilator | 1.13 | 0.99 - 1.27 | 0.087 |
| **Age** |  |  |  |
| 21-35 | Reference | -- | -- |
| 36-45 | 1.24 | 1.15 - 1.33 | <0.001 |
| 46-65 | 1.32 | 1.23 - 1.41 | 0 |
| 66+ | 1.30 | 1.21 - 1.38 | 0 |
| **Male Sex** | 0.66 | 0.60 - 0.72 | 0 |
| **Race/ethnicity**^2^ |  |  |  |
| Non-Hispanic Asian | 0.92 | 0.74 - 1.10 | 0.371 |
| Non-Hispanic Black | 0.84 | 0.76 - 0.93 | <0.001 |
| Non-Hispanic White | Reference | -- | -- |
| Non-Hispanic Other/Multiple | 1.03 | 0.90 - 1.15 | 0.656 |
| Hispanic or Latino | 0.98 | 0.89 - 1.06 | 0.625 |
| Missing/unknown | 0.75 | 0.647 - 0.850 | 0 |
| **Charlson comorbidity index ^3^** |  |  |  |
| 0 | Reference | -- | -- |
| 1-3 | 1.28 | 1.21 - 1.35 | 0 |
| 4+ | 1.42 | 1.33 - 1.52 | 0 |
| **Smoking status** |  |  |  |
| Never | Reference | -- | -- |
| Ever | 0.92 | 0.84 - 0.99 | 0.026 |
| Missing/Unknown | 0.918 | 0.85 - 0.99 | 0.017 |
| **Prior substance abuse** | 1.38 | 1.28 - 1.49 | 0 |
| **Body mass index** ^4^ |  |  |  |
| underweight | 0.97 | 0.65 - 1.28 | 0.831 |
| normal weight | Reference | -- | -- |
| overweight | 1.05 | 0.95 - 1.15 | 0.317 |
| moderately obese | 1.00 | 0.90 - 1.10 | 0.979 |
| severely obese | 1.01 | 0.89 - 1.13 | 0.853 |
| morbidly obese | 1.00 | 0.88 - 1.13 | 0.941 |
| missing/unknown | 0.845 | 0.74 - 0.95 | 0.002 |
| **Prior glucocorticoid use** ^5^ | 1.24 | 1.16 - 1.32 | <0.001 |
| **Medical insurance** |  |  |  |
| Private | Reference | -- | -- |
| Medicare/Medicaid | 0.97 | 0.88 - 1.06 | 0.498 |
| Other | 0.38 | 0.01 - 0.74 | <0.001 |
| Missing/Unknown | 0.89 | 0.82 - 0.96 | 0.002 |
| **RECOVER site size**^7^ |  |  |  |
| Quartile 1 | Reference | -- | -- |
| Quartile 2 | 1.38 | 0.96 - 1.79 | 0.132 |
| Quartile 3 | 1.30 | 0.89 - 1.71 | 0.209 |
| Quartile 4 | 1.47 | 1.07 - 1.88 | 0.061 |
| **Prior encounters** ^6^ |  |  |  |
| 1-2 | Reference | -- | -- |
| 0 | 0.63 | 0.52 - 0.74 | 0 |
| 3-5 | 1.17 | 1.06 - 1.27 | 0.004 |
| 6-10 | 1.37 | 1.26 - 1.47 | 0 |
| 11-20 | 1.62 | 1.52 - 1.73 | 0 |
| 21+ | 2.10 | 1.99 - 2.20 | 0 |
| **Index date period** |  |  |  |
| Apr-June 2020 | Reference | -- | -- |
| Jul-Sep 2020 | 0.95 | 0.86 - 1.04 | 0.233 |
| Oct-Dec 2020 | 0.89 | 0.81 - 0.97 | 0.004 |
| Jan -Apr 2021 | 0.89 | 0.81 - 0.98 | 0.009 |
| **Model 6:** Full adjustment^8^- no prior^9^ & sex-stratified (male) | | | |
| **COVID-19 severity** |  |  |  |
| Outpatient | Reference | -- | -- |
| Emergency department | 0.89 | 0.73 - 1.05 | 0.140 |
| Hospitalized | 0.94 | 0.78 - 1.09 | 0.406 |
| Hospitalized and on ventilator | 1.26 | 1.05 - 1.47 | 0.023 |
| **Age** |  |  |  |
| 21-35 | Reference | -- | -- |
| 36-45 | 1.18 | 1.00 - 1.36 | 0.072 |
| 46-65 | 1.27 | 1.10 - 1.44 | 0.007 |
| 66+ | 1.37 | 1.21 - 1.52 | <0.001 |
| **Race/ethnicity**^2^ |  |  |  |
| Non-Hispanic Asian | 0.87 | 0.55 - 1.19 | 0.406 |
| Non-Hispanic Black | 0.87 | 0.72 - 1.02 | 0.075 |
| Non-Hispanic White | Reference | -- | -- |
| Non-Hispanic Other/Multiple | 1.05 | 0.84 - 1.26 | 0.659 |
| Hispanic or Latino | 0.93 | 0.78 - 1.08 | 0.332 |
| Missing/unknown | 0.71 | 0.54 - 0.88 | <0.001 |
| **Charlson comorbidity index ^3^** |  |  |  |
| 0 | Reference | -- | -- |
| 1-3 | 1.43 | 1.31 - 1.56 | 0 |
| 4+ | 1.55 | 1.39 - 1.72 | <0.001 |
| **Smoking status** |  |  |  |
| Never | Reference | -- | -- |
| Ever | 0.92 | 0.79 - 1.04 | 0.168 |
| Missing/Unknown | 0.86 | 0.73 - 0.98 | 0.013 |
| **Prior substance abuse** | 1.44 | 1.29 - 1.59 | <0.001 |
| **Body mass index** ^4^ |  |  |  |
| underweight | 0.28 | 0.86 - 1.42 | 0.029 |
| normal weight | Reference | -- | -- |
| overweight | 0.95 | 0.78 - 1.12 | 0.558 |
| moderately obese | 0.92 | 0.74 - 1.10 | 0.370 |
| severely obese | 0.94 | 0.72 - 1.15 | 0.540 |
| morbidly obese | 0.83 | 0.58 - 1.08 | 0.136 |
| missing/unknown | 0.86 | 0.67 - 1.05 | 0.119 |
| **Prior glucocorticoid use** ^5^ | 1.17 | 1.02 - 1.31 | 0.039 |
| **Medical insurance** |  |  |  |
| Private | Reference | -- | -- |
| Medicare/Medicaid | 1.02 | 0.86 - 1.18 | 0.840 |
| Other | 0.59 | 0.12 - 1.07 | 0.03 |
| Missing/Unknown | 0.95 | 0.82 - 1.08 | 0.43 |
| **RECOVER site size**^7^ |  |  |  |
| Quartile 1 | Reference | -- | -- |
| Quartile 2 | 1.28 | 0.56 - 2.00 | 0.50 |
| Quartile 3 | 1.38 | 0.67 - 2.09 | 0.371 |
| Quartile 4 | 1.60 | 0.90 - 2.30 | 0.191 |
| **Prior encounters** ^6^ |  |  |  |
| 1-2 | Reference | -- | -- |
| 0 | 0.56 | 0.38 - 0.74 | 0 |
| 3-5 | 1.21 | 1.03 - 1.39 | 0.033 |
| 6-10 | 1.50 | 1.32 - 1.68 | <0.001 |
| 11-20 | 1.81 | 1.62 - 1.99 | 0 |
| 21+ | 2.22 | 2.02 - 2.41 | 0 |
| **Index date period** |  |  |  |
| Apr-June 2020 | Reference | -- | -- |
| Jul-Sep 2020 | 1.07 | 0.91 - 1.22 | 0.425 |
| Oct-Dec 2020 | 0.99 | 0.85 - 1.13 | 0.882 |
| Jan -Apr 2021 | 0.927 | 0.781 - 1.07 | 0.310 |
| **Model 7:** Full adjustment^8^- no prior^9^ & sex-stratified (female) | | | |
| **COVID severity** |  |  |  |
| Outpatient | Reference | -- | -- |
| Emergency department | 0.90 | 0.80 - 1.01 | 0.061 |
| Hospitalized | 0.74 | 0.61 - 0.87 | <0.001 |
| Hospitalized and on ventilator | 1.04 | 0.85 - 1.23 | <0.001 |
| **Age** |  |  |  |
| 21-35 | Reference | -- | -- |
| 36-45 | 1.26 | 1.16 - 1.37 | <0.001 |
| 46-65 | 1.35 | 1.24 - 1.46 | 0 |
| 66+ | 1.25 | 1.15- 1.35 | <0.001 |
| **Race/ethnicity**^2^ |  |  |  |
| Non-Hispanic Asian | 0.94 | 0.72 - 1.16 | 0.603 |
| Non-Hispanic Black | 0.83 | 0.73 - 0.93 | <0.001 |
| Non-Hispanic White | Reference | -- | -- |
| Non-Hispanic Other/Multiple | 1.02 | 0.86 - 1.18 | 0.816 |
| Hispanic or Latino | 1.01 | 0.90 - 1.11 | 0.911 |
| Missing/unknown | 0.78 | 0.65 - 0.90 | <0.001 |
| **Charlson comorbidity index ^3^** |  |  |  |
| 0 | Reference | -- | -- |
| 1-3 | 1.21 | 1.13 - 1.30 | <0.001 |
| 4+ | 1.34 | 1.22 - 1.46 | <0.001 |
| **Smoking status** |  |  |  |
| Never | Reference | -- | -- |
| Ever | 0.91 | 0.82 - 1.01 | 0.056 |
| Missing/Unknown | 0.95 | 0.87 - 1.04 | 0.273 |
| **Prior substance abuse** | 1.32 | 1.18 - 1.46 | <0.001 |
| **Body mass index** ^4^ |  |  |  |
| underweight | 1.23 | 0.90 - 1.56 | 0.225 |
| normal weight | Reference | -- | -- |
| overweight | 1.10 | 0.99 - 1.22 | 0.102 |
| moderately obese | 1.04 | 0.92 - 1.17 | 0.53 |
| severely obese | 1.06 | 0.92 - 1.20 | 0.439 |
| morbidly obese | 1.09 | 0.95 - 1.23 | 0.216 |
| missing/unknown | 0.835 | 0.71 - 0.96 | 0.006 |
| **Prior glucocorticoid use** ^5^ | 1.28 | 1.19 - 1.38 | <0.001 |
| **Medical insurance** |  |  |  |
| Private | Reference | -- | -- |
| Medicare/Medicaid | 0.95 | 0.84 - 1.06 | 0.345 |
| Other | 0.234 | 0.20-0.83 | <0.001 |
| Missing/Unknown | 0.86 | 0.77 - 0.95 | 0.001 |
| **RECOVER site size**^7^ |  |  |  |
| Quartile 1 | Reference | -- | -- |
| Quartile 2 | 1.44 | 0.94 - 1.95 | 0.156 |
| Quartile 3 | 1.28 | 0.77 - 1.78 | 0.342 |
| Quartile 4 | 1.43 | 0.94 - 1.93 | 0.155 |
| **Prior encounters** ^6^ |  |  |  |
| 1-2 | Reference | -- | -- |
| 0 | 0.69 | 0.55 - 0.82 | <0.001 |
| 3-5 | 1.13 | 1.01 - 1.26 | 0.056 |
| 6-10 | 1.29 | 1.17 - 1.42 | <0.001 |
| 11-20 | 1.53 | 1.40 - 1.66 | 0 |
| 21+ | 2.03 | 1.89 - 2.16 | 0 |
| **Index date period** |  |  |  |
| Apr-June 2020 | Reference | -- | -- |
| Jul-Sep 2020 | 0.89 | 0.78 - 1.00 | 0.046 |
| Oct-Dec 2020 | 0.84 | 0.75 - 0.94 | 0.001 |
| Jan -Apr 2021 | 0.88 | 0.78 - 0.98 | 0.014 |

Abbreviation: SD, standard deviation

(1) History of prior autoimmune disease definition: Patients with at least 1 autoimmune disease ICD-9/ICD-10/SNOMED code within any of the autoimmune disease concept set at any point prior to their index date.

(2) Race/ethnicity is commonly inconsistent and/or inaccurately captured in the EHR, especially among minorities, and can lead to significant missing data and misclassification biases (31, 32). Hispanic ethnicity was defined as its own category and not cross-reported with the other race categories for ease of standardization and comparison across a large network of health systems.

(3) Charlson Comorbidity Index, CCI, is a weighted index classification system using a collection of EHR diagnoses codes from all available data prior to the patient’s SARS-CoV-2 infection index to define a patient’s risk of mortality (25).

(4) Body Mass Index (BMI, in kg/m^2^) is categorized according to the World Health Organization (WHO) and National Institute of Health (NIH) standards. Categorization BMI cut offs are as follows: Underweight: < 18.5 kg/m^2^, normal: 18.5 to 24.9 kg/m^2,^ overweight: 25 to 29.9 kg/m^2,^ moderately obese: 30-34.9 kg/m^2^**,** severely obese: 35-39.9 kg/m^2,^ morbidly obese**:** ≥ 40 (27**).** The most recent recorded BMI, either on or up to 1- year prior SARS-CoV-2 infection index event, was used.

(5) Prior glucocorticoid use was defined by having at least 1 prescription for oral dexamethasone, betamethasone, prednisolone, methylprednisolone, triamcinolone, hydrocortisone, prednisone, or triamcinolone within 1 year to 1 week prior to SARS-CoV-2 infection index event. See S6 appendix.

(6) Prior encounters were defined as the number of prior billing encounters, calculated as unique days and assuming each outpatient and ED visit is 1 day, in a patient's EHR in the 1 year before SARS-CoV-2 infection index event.

(7) RECOVER site quartiles are based on overall RECOVER patient enrollment numbers. Sites are categorized by the following; quartile 1: <140,000 participants, quartile 2: <220,000 participants, quartile 3: <400,000 participants, quartile 4: ≥ 400,000 participants

(8) Full model adjusted for age, sex, race, medical insurance type, healthcare utilization, date of COVID-19 infection, comorbidities, substance use disorder, smoking status (adults only), body mass index glucocorticoid use, and RECOVER enrollment site size

(9): No prior indicated models excluding those with a history of autoimmune disease
