# Supplementary material for: Severity of acute SARS-CoV-2 infection and risk of new-onset autoimmune disease: A RECOVER initiative study in nationwide U.S. cohorts
Source: PLoS One. 2025 Jun 4;20(6):e0324513. doi: 10.1371/journal.pone.0324513 (PMC12136303; doi:10.1371/journal.pone.0324513)
Supplement: S4 Table — (DOCX) [file pone.0324513.s013.docx]

|  | **aHR** | **95% CI** | **p-value** |
| --- | --- | --- | --- |
| **Model 1:** Age, sex, and race/ethnicity | | | |
| **COVID severity** |  |  |  |
| Asymptomatic | Reference | -- | -- |
| Mild | 1.07 | 0.92-1.25 | 0.366 |
| Moderate | 3.65 | 2.84-4.69 | <0.001 |
| Severe | 6.93 | 5.49-8.73 | <0.001 |
| **Age** |  |  |  |
| <1 | Reference | -- | -- |
| 1 to 4 | 1.25 | 0.82-1.89 | 0.296 |
| 5 to 9 | 1.78 | 1.20-2.65 | 0.005 |
| 10 to 15 | 2.49 | 1.71-3.63 | <0.001 |
| 16 to 20 | 2.18 | 1.50-3.18 | <0.001 |
| **Male Sex** | 0.82 | 0.72-0.94 | 0.004 |
| **Race/ethnicity**^2^ |  |  |  |
| Non-Hispanic Asian | 0.85 | 0.54-1.31 | 0.453 |
| Non-Hispanic Black | 0.66 | 0.53-0.82 | <0.001 |
| Non-Hispanic White | Reference | -- | -- |
| Non-Hispanic Other/Multiple | 1.23 | 0.77-1.95 | 0.382 |
| Hispanic or Latino | 0.84 | 0.72-0.99 | 0.032 |
| Missing/unknown | 0.61 | 0.49-0.78 | <0.001 |
| **Model 2:** Full adjustment^9^ | | | |
| **COVID severity** |  |  |  |
| Asymptomatic | Reference | -- | -- |
| Mild | 1.04 | 0.89-1.22 | 0.609 |
| Moderate | 2.15 | 1.65-2.79 | <0.001 |
| Severe | 3.14 | 2.42-4.07 | <0.001 |
| **Age** |  |  |  |
| <1 | Reference | -- | -- |
| 1 to 4 | 1.08 | 0.71-1.65 | 0.724 |
| 5 to 9 | 1.65 | 1.08-2.51 | 0.020 |
| 10 to 15 | 2.33 | 1.56-3.48 | <0.001 |
| 16 to 20 | 1.97 | 1.33-2.93 | <0.001 |
| **Male Sex** | 0.83 | 0.73-0.96 | 0.009 |
| **Race/ethnicity**^2^ |  |  |  |
| Non-Hispanic Asian | 0.83 | 0.53-1.29 | 0.405 |
| Non-Hispanic Black | 0.66 | 0.52-0.82 | <0.001 |
| Non-Hispanic White | Reference | -- | -- |
| Non-Hispanic Other/Multiple | 1.10 | 0.69-1.75 | 0.680 |
| Hispanic or Latino | 0.91 | 0.76-1.07 | 0.254 |
| Missing/unknown | 0.67 | 0.53-0.85 | 0.001 |
| **Pediatric medical complexity algorithm: complex/chronic** ^8^ |  |  |  |
| Neither chronic nor complex | Reference | -- | -- |
| Chronic | 1.00 | 0.75-1.34 | 0.975 |
| Complex-chronic | 1.54 | 1.24-1.90 | <0.001 |
| **Prior substance abuse** | 0.85 | 0.56-1.29 | 0.447 |
| **Body mass index percentile** ^4^ |  |  |  |
| Below 5^th^ percentile | 1.46 | 1.02-2.08 | 0.037 |
| 5^th^ – 84^th^ percentile | 1.38 | 1.14-1.66 | <0.001 |
| 85^th^ – 94^th^ percentile | 1.16 | 0.88-1.53 | 0.294 |
| Above 95^th^ percentile | 1.20 | 0.98-1.48 | 0.075 |
| missing/unknown | Reference | -- | -- |
| **Prior glucocorticoid use** ^5^ | 1.45 | 1.21-1.75 | <0.001 |
| **Medical insurance** |  |  |  |
| Private | Reference | -- | -- |
| Medicare/Medicaid | 1.19 | 0.93-1.54 | 0.174 |
| Other | 0.00 | 0.00-Inf | 0.984 |
| Missing/Unknown | 0.92 | 0.75-1.12 | 0.405 |
| **Prior encounters** ^6^ |  |  |  |
| 0 | Reference | -- | -- |
| 1 to 2 | 1.19 | 0.86-1.64 | 0.295 |
| 3 to 5 | 1.65 | 1.19-2.29 | 0.003 |
| 6 to 10 | 1.90 | 1.35-2.66 | <0.001 |
| 11 to 20 | 2.61 | 1.85-3.68 | <0.001 |
| 21+ | 3.43 | 2.39-4.90 | <0.001 |
| **Index date period** |  |  |  |
| Apr-June 2020 | Reference | -- | -- |
| Jul-Sep 2020 | 0.77 | 0.60-1.00 | 0.051 |
| Oct-Dec 2020 | 0.71 | 0.56-0.90 | 0.005 |
| Jan-Apr 2021 | 0.78 | 0.61-0.99 | 0.039 |
| **RECOVER site size**^7^ |  |  |  |
| Quartile 1 | Reference | -- | -- |
| Quartile 2 | 0.86 | 0.67-1.10 | 0.215 |
| Quartile 3 | 0.92 | 0.72-1.18 | 0.527 |
| Quartile 4 | 0.60 | 0.48-0.75 | <0.001 |
| **Model 3:** Full adjustment^9^- sex-stratified (male) | | | |
| **COVID severity** |  |  |  |
| Asymptomatic | Reference | -- | -- |
| Mild | 1.01 | 0.80-1.28 | 0.924 |
| Moderate | 2.19 | 1.47-3.26 | <0.001 |
| Severe | 3.08 | 2.11-4.50 | <0.001 |
| **Age** |  |  |  |
| <1 | Reference | -- | -- |
| 1 to 4 | 1.22 | 0.67-2.21 | 0.508 |
| 5 to 9 | 1.84 | 1.01-3.33 | 0.045 |
| 10 to 15 | 2.38 | 1.34-4.22 | 0.003 |
| 16 to 20 | 1.97 | 1.11-3.50 | 0.020 |
| **Race/ethnicity**^2^ |  |  |  |
| Non-Hispanic Asian | 0.81 | 0.44-1.49 | 0.497 |
| Non-Hispanic Black | 0.58 | 0.41-0.81 | 0.002 |
| Non-Hispanic White | Reference | -- | -- |
| Non-Hispanic Other/Multiple | 0.81 | 0.38-1.73 | 0.589 |
| Hispanic or Latino | 0.81 | 0.63-1.04 | 0.102 |
| Missing/unknown | 0.47 | 0.31-0.69 | <0.001 |
| **Pediatric medical complexity algorithm: complex/chronic** ^8^ |  |  |  |
| Neither chronic nor complex | Reference | -- | -- |
| Chronic | 0.69 | 0.43-1.11 | 0.124 |
| Complex-chronic | 1.42 | 1.03-1.95 | 0.030 |
| **Prior substance abuse** | 1.00 | 0.55-1.85 | 0.988 |
| **Body mass index percentile** ^4^ |  |  |  |
| Below 5^th^ percentile | 1.71 | 1.06-2.77 | 0.027 |
| 5^th^ – 84^th^ percentile | 1.32 | 0.99-1.76 | 0.058 |
| 85^th^ – 94^th^ percentile | 1.37 | 0.92-2.06 | 0.125 |
| Above 95^th^ percentile | 1.25 | 0.92-1.70 | 0.160 |
| missing/unknown | Reference | -- | -- |
| **Prior glucocorticoid use** ^5^ | 1.45 | 1.10-1.91 | 0.009 |
| **Medical insurance** |  |  |  |
| Private | Reference | -- | -- |
| Medicare/Medicaid | 1.20 | 0.84-1.72 | 0.327 |
| Other | 0.00 | 0.00-Inf | 0.989 |
| Missing/Unknown | 0.79 | 0.59-1.06 | 0.121 |
| **Prior encounters** ^6^ |  |  |  |
| 0 | Reference | -- | -- |
| 1 to 2 | 1.18 | 0.73-1.93 | 0.499 |
| 3 to 5 | 1.76 | 1.08-2.88 | 0.024 |
| 6 to 10 | 2.23 | 1.35-3.70 | 0.002 |
| 11 to 20 | 2.57 | 1.52-4.37 | 0.001 |
| 21+ | 3.74 | 2.17-6.43 | <0.001 |
| **Index date period** |  |  |  |
| Apr-June 2020 | Reference | -- | -- |
| Jul-Sep 2020 | 1.20 | 0.79-1.82 | 0.402 |
| Oct-Dec 2020 | 1.01 | 0.68-1.50 | 0.947 |
| Jan-Apr 2021 | 0.96 | 0.64-1.44 | 0.855 |
| **RECOVER site size**^7^ |  |  |  |
| Quartile 1 | Reference | -- | -- |
| Quartile 2 | 0.90 | 0.61-1.33 | 0.607 |
| Quartile 3 | 1.11 | 0.76-1.63 | 0.582 |
| Quartile 4 | 0.62 | 0.43-0.88 | 0.009 |
| **Model 4:** Full adjustment^9^- sex-stratified (female) | | | |
| **COVID severity** |  |  |  |
| Asymptomatic | Reference | -- | -- |
| Mild | 1.06 | 0.87-1.31 | 0.551 |
| Moderate | 2.09 | 1.47-2.96 | <0.001 |
| Severe | 3.23 | 2.27-4.60 | <0.001 |
| **Age** |  |  |  |
| <1 | Reference | -- | -- |
| 1 to 4 | 0.93 | 0.51-1.71 | 0.813 |
| 5 to 9 | 1.46 | 0.81-2.64 | 0.213 |
| 10 to 15 | 2.28 | 1.31-3.99 | 0.004 |
| 16 to 20 | 1.93 | 1.12-3.35 | 0.019 |
| **Race/ethnicity**^2^ |  |  |  |
| Non-Hispanic Asian | 0.82 | 0.43-1.54 | 0.530 |
| Non-Hispanic Black | 0.72 | 0.54-0.97 | 0.031 |
| Non-Hispanic White | Reference | -- | -- |
| Non-Hispanic Other/Multiple | 1.37 | 0.76-2.47 | 0.289 |
| Hispanic or Latino | 0.98 | 0.78-1.23 | 0.870 |
| Missing/unknown | 0.87 | 0.64-1.17 | 0.352 |
| **Pediatric medical complexity algorithm: complex/chronic** ^8^ |  |  |  |
| Neither chronic nor complex | Reference | -- | -- |
| Chronic | 1.33 | 0.93-1.90 | 0.122 |
| Complex-chronic | 1.65 | 1.24-2.19 | 0.001 |
| **Prior substance abuse** | 0.74 | 0.43-1.30 | 0.302 |
| **Body mass index percentile** ^4^ |  |  |  |
| Below 5^th^ percentile | 1.23 | 0.72-2.08 | 0.454 |
| 5^th^ – 84^th^ percentile | 1.43 | 1.12-1.83 | 0.005 |
| 85^th^ – 94^th^ percentile | 1.02 | 0.70-1.48 | 0.928 |
| Above 95^th^ percentile | 1.17 | 0.89-1.53 | 0.263 |
| missing/unknown | Reference | -- | -- |
| **Prior glucocorticoid use** ^5^ | 1.46 | 1.14-1.88 | 0.003 |
| **Medical insurance** |  |  |  |
| Private | Reference | -- | -- |
| Medicare/Medicaid | 1.19 | 0.83-1.70 | 0.348 |
| Other | 0.00 | 0.00-Inf | 0.983 |
| Missing/Unknown | 1.04 | 0.79-1.39 | 0.762 |
| **Prior encounters** ^6^ |  |  |  |
| 0 | Reference | -- | -- |
| 1 to 2 | 1.19 | 0.77-1.82 | 0.434 |
| 3 to 5 | 1.58 | 1.02-2.44 | 0.041 |
| 6 to 10 | 1.66 | 1.06-2.61 | 0.028 |
| 11 to 20 | 2.61 | 1.66-4.12 | <0.001 |
| 21+ | 3.16 | 1.96-5.11 | <0.001 |
| **Index date period** |  |  |  |
| Apr-June 2020 | Reference | -- | -- |
| Jul-Sep 2020 | 0.57 | 0.41-0.80 | 0.001 |
| Oct-Dec 2020 | 0.56 | 0.42-0.76 | <0.001 |
| Jan-Apr 2021 | 0.68 | 0.51-0.92 | 0.013 |
| **RECOVER site size**^7^ |  |  |  |
| Quartile 1 | Reference | -- | -- |
| Quartile 2 | 0.82 | 0.59-1.12 | 0.207 |
| Quartile 3 | 0.78 | 0.57-1.09 | 0.143 |
| Quartile 4 | 0.58 | 0.43-0.79 | <0.001 |
| **Model 5:** Full adjustment^9^- no prior^10^ | | | |
| **COVID severity** |  |  |  |
| Asymptomatic | Reference | -- | -- |
| Mild | 1.02 | 0.87-1.20 | 0.786 |
| Moderate | 2.19 | 1.64-2.93 | <0.001 |
| Severe | 3.29 | 2.47-4.39 | <0.001 |
| **Age** |  |  |  |
| <1 | Reference | -- | -- |
| 1 to 4 | 1.07 | 0.69-1.63 | 0.773 |
| 5 to 9 | 1.59 | 1.04-2.43 | 0.033 |
| 10 to 15 | 2.35 | 1.57-3.52 | <0.001 |
| 16 to 20 | 1.90 | 1.27-2.84 | 0.002 |
| **Male Sex** | 0.88 | 0.77-1.02 | 0.094 |
| **Race/ethnicity**^2^ |  |  |  |
| Non-Hispanic Asian | 0.89 | 0.56-1.39 | 0.599 |
| Non-Hispanic Black | 0.64 | 0.50-0.81 | <0.001 |
| Non-Hispanic White | Reference | -- | -- |
| Non-Hispanic Other/Multiple | 1.20 | 0.75-1.94 | 0.448 |
| Hispanic or Latino | 0.92 | 0.76-1.10 | 0.351 |
| Missing/unknown | 0.68 | 0.53-0.88 | 0.003 |
| **Pediatric medical complexity algorithm: complex/chronic** ^8^ |  |  |  |
| Neither chronic nor complex | Reference | -- | -- |
| Chronic | 0.90 | 0.66-1.24 | 0.537 |
| Complex-chronic | 1.39 | 1.10-1.76 | 0.006 |
| **Prior substance abuse** | 0.88 | 0.56-1.39 | 0.591 |
| **Body mass index percentile** ^4^ |  |  |  |
| Below 5^th^ percentile | 1.41 | 0.96-2.08 | 0.080 |
| 5^th^ – 84^th^ percentile | 1.39 | 1.14-1.69 | 0.001 |
| 85^th^ – 94^th^ percentile | 1.12 | 0.83-1.51 | 0.469 |
| Above 95^th^ percentile | 1.21 | 0.98-1.51 | 0.077 |
| missing/unknown | Reference | -- | -- |
| **Prior glucocorticoid use** ^5^ | 1.38 | 1.12-1.69 | 0.002 |
| **Medical insurance** |  |  |  |
| Private | Reference | -- | -- |
| Medicare/Medicaid | 1.21 | 0.92-1.58 | 0.172 |
| Other | 0.00 | 0.00-Inf | 0.985 |
| Missing/Unknown | 0.87 | 0.70-1.08 | 0.207 |
| **Mean prior encounters** ^6^ |  |  |  |
| 0 | Reference | -- | -- |
| 1 to 2 | 1.14 | 0.82-1.59 | 0.433 |
| 3 to 5 | 1.60 | 1.14-2.23 | 0.007 |
| 6 to 10 | 1.99 | 1.41-2.81 | <0.001 |
| 11 to 20 | 2.65 | 1.85-3.78 | <0.001 |
| 21+ | 3.46 | 2.37-5.03 | <0.001 |
| **Index date period** |  |  |  |
| Apr-June 2020 | Reference | -- | -- |
| Jul-Sep 2020 | 0.76 | 0.58-1.00 | 0.049 |
| Oct-Dec 2020 | 0.71 | 0.55-0.91 | 0.007 |
| Jan-Apr 2021 | 0.79 | 0.61-1.02 | 0.072 |
| **RECOVER site size**^7^ |  |  |  |
| Quartile 1 | Reference | -- | -- |
| Quartile 2 | 0.87 | 0.67-1.13 | 0.293 |
| Quartile 3 | 0.94 | 0.72-1.22 | 0.637 |
| Quartile 4 | 0.59 | 0.46-0.75 | <0.001 |
| **Model 6:** Full adjustment^9^- no prior^10^ & sex-stratified (male) | | | |
| **COVID severity** |  |  |  |
| Asymptomatic | Reference | -- | -- |
| Mild | 1.00 | 0.78-1.28 | 0.986 |
| Moderate | 2.24 | 1.46-3.42 | <0.001 |
| Severe | 3.31 | 2.21-4.96 | <0.001 |
| **Age** |  |  |  |
| <1 | Reference | -- | -- |
| 1 to 4 | 1.26 | 0.69-2.28 | 0.452 |
| 5 to 9 | 1.80 | 0.99-3.29 | 0.055 |
| 10 to 15 | 2.46 | 1.38-4.38 | 0.002 |
| 16 to 20 | 2.00 | 1.13-3.57 | 0.018 |
| **Race/ethnicity**^2^ |  |  |  |
| Non-Hispanic Asian | 0.93 | 0.50-1.71 | 0.806 |
| Non-Hispanic Black | 0.58 | 0.41-0.84 | 0.004 |
| Non-Hispanic White | Reference | -- | -- |
| Non-Hispanic Other/Multiple | 0.96 | 0.45-2.06 | 0.916 |
| Hispanic or Latino | 0.88 | 0.67-1.15 | 0.347 |
| Missing/unknown | 0.52 | 0.35-0.78 | 0.001 |
| **Pediatric medical complexity algorithm: complex/chronic** ^8^ |  |  |  |
| Neither chronic nor complex | Reference | -- | -- |
| Chronic | 0.63 | 0.38-1.06 | 0.081 |
| Complex-chronic | 1.37 | 0.98-1.92 | 0.069 |
| **Prior substance abuse** | 0.95 | 0.49-1.87 | 0.888 |
| **Body mass index percentile** ^4^ |  |  |  |
| Below 5^th^ percentile | 1.49 | 0.88-2.54 | 0.138 |
| 5^th^ – 84^th^ percentile | 1.31 | 0.97-1.76 | 0.076 |
| 85^th^ – 94^th^ percentile | 1.29 | 0.84-1.98 | 0.247 |
| Above 95^th^ percentile | 1.27 | 0.93-1.74 | 0.139 |
| missing/unknown | Reference | -- | -- |
| **Prior glucocorticoid use** ^5^ | 1.43 | 1.07-1.92 | 0.017 |
| **Medical insurance** |  |  |  |
| Private | Reference | -- | -- |
| Medicare/Medicaid | 1.08 | 0.74-1.58 | 0.695 |
| Other | 0.00 | 0.00-Inf | 0.989 |
| Missing/Unknown | 0.73 | 0.54-0.99 | 0.043 |
| **Prior encounters** ^6^ |  |  |  |
| 0 | Reference | -- | -- |
| 1 to 2 | 1.11 | 0.68-1.82 | 0.670 |
| 3 to 5 | 1.68 | 1.02-2.76 | 0.041 |
| 6 to 10 | 2.32 | 1.40-3.84 | 0.001 |
| 11 to 20 | 2.63 | 1.54-4.49 | <0.001 |
| 21+ | 3.68 | 2.11-6.43 | <0.001 |
| **Index date period** |  |  |  |
| Apr-June 2020 | Reference | -- | -- |
| Jul-Sep 2020 | 1.17 | 0.76-1.80 | 0.468 |
| Oct-Dec 2020 | 0.92 | 0.61-1.38 | 0.687 |
| Jan-Apr 2021 | 0.95 | 0.63-1.44 | 0.815 |
| **RECOVER site size**^7^ |  |  |  |
| Quartile 1 | Reference | -- | -- |
| Quartile 2 | 0.88 | 0.59-1.32 | 0.547 |
| Quartile 3 | 1.02 | 0.69-1.51 | 0.919 |
| Quartile 4 | 0.55 | 0.38-0.80 | 0.002 |
| **Model 7:** Full adjustment^9^- no prior^10^ & sex-stratified (female) | | | |
| **COVID severity** |  |  |  |
| Asymptomatic | Reference | -- | -- |
| Mild | 1.04 | 0.84-1.30 | 0.699 |
| Moderate | 2.11 | 1.42-3.14 | <0.001 |
| Severe | 3.36 | 2.24-5.05 | <0.001 |
| **Age** |  |  |  |
| <1 | Reference | -- | -- |
| 1 to 4 | 0.87 | 0.47-1.62 | 0.657 |
| 5 to 9 | 1.37 | 0.75-2.51 | 0.304 |
| 10 to 15 | 2.24 | 1.27-3.95 | 0.005 |
| 16 to 20 | 1.79 | 1.03-3.13 | 0.040 |
| **Race/ethnicity**^2^ |  |  |  |
| Non-Hispanic Asian | 0.81 | 0.42-1.60 | 0.550 |
| Non-Hispanic Black | 0.69 | 0.50-0.95 | 0.024 |
| Non-Hispanic White | Reference | -- | -- |
| Non-Hispanic Other/Multiple | 1.42 | 0.77-2.63 | 0.262 |
| Hispanic or Latino | 0.94 | 0.74-1.21 | 0.652 |
| Missing/unknown | 0.83 | 0.60-1.16 | 0.276 |
| **Pediatric medical complexity algorithm: complex/chronic** ^8^ |  |  |  |
| Neither chronic nor complex | Reference | -- | -- |
| Chronic | 1.21 | 0.80-1.81 | 0.367 |
| Complex-chronic | 1.40 | 1.01-1.95 | 0.043 |
| **Prior substance abuse** | 0.82 | 0.45-1.52 | 0.534 |
| **Body mass index percentile** ^4^ |  |  |  |
| Below 5^th^ percentile | 1.34 | 0.76-2.37 | 0.307 |
| 5^th^ – 84^th^ percentile | 1.47 | 1.13-1.91 | 0.005 |
| 85^th^ – 94^th^ percentile | 0.99 | 0.65-1.49 | 0.948 |
| Above 95^th^ percentile | 1.16 | 0.87-1.56 | 0.314 |
| missing/unknown | Reference | -- | -- |
| **Prior glucocorticoid use** ^5^ | 1.33 | 1.00-1.77 | 0.052 |
| **Medical insurance** |  |  |  |
| Private | Reference | -- | -- |
| Medicare/Medicaid | 1.35 | 0.92-1.98 | 0.128 |
| Other | 0.00 | 0.00-Inf | 0.984 |
| Missing/Unknown | 1.03 | 0.75-1.40 | 0.871 |
| **Prior encounters** ^6^ |  |  |  |
| 0 | Reference | -- | -- |
| 1 to 2 | 1.16 | 0.74-1.81 | 0.521 |
| 3 to 5 | 1.53 | 0.97-2.42 | 0.068 |
| 6 to 10 | 1.75 | 1.09-2.80 | 0.020 |
| 11 to 20 | 2.64 | 1.64-4.26 | <0.001 |
| 21+ | 3.24 | 1.94-5.40 | <0.001 |
| **Index date period** |  |  |  |
| Apr-June 2020 | Reference | -- | -- |
| Jul-Sep 2020 | 0.53 | 0.37-0.77 | 0.001 |
| Oct-Dec 2020 | 0.58 | 0.42-0.80 | 0.001 |
| Jan-Apr 2021 | 0.70 | 0.50-0.97 | 0.030 |
| **RECOVER site size**^7^ |  |  |  |
| Quartile 1 | Reference | -- | -- |
| Quartile 2 | 0.85 | 0.60-1.21 | 0.359 |
| Quartile 3 | 0.86 | 0.60-1.22 | 0.390 |
| Quartile 4 | 0.61 | 0.44-0.85 | 0.003 |

Abbreviation: SD, standard deviation

(1) History of prior autoimmune disease definition: Patients with at least 1 autoimmune disease ICD-9/ICD-10/SNOMED code within any of the autoimmune disease concept set at any point prior to their index date.

(2) Race/ethnicity is commonly inconsistent and/or inaccurately captured in the EHR, especially among minorities, and can lead to significant missing data and misclassification biases (31, 32). Hispanic ethnicity was defined as its own category and not cross-reported with the other race categories for ease of standardization and comparison across a large network of health systems.

(4) Body Mass Index (BMI in kg/m^2^) is categorized according to the World Health Organization (WHO) and National Institute of Health (NIH) standards. Categorized BMI cut-offs for adults are as follows: Underweight: < 18.5 kg/m^2^, normal: 18.5 to 24.9 kg/m^2,^ overweight: 25 to 29.9 kg/m^2,^ moderately obese: 30-34.9 kg/m^2^**,** severely obese: 35-39.9 kg/m^2,^ morbidly obese**:** ≥ 40 (27)**.** The most recent recorded BMI, either on or up to 1- year prior to the SARS-CoV-2 infection index event, was used.

(5) Prior glucocorticoid use was defined by having at least 1 prescription for oral dexamethasone, betamethasone, prednisolone, methylprednisolone, triamcinolone, hydrocortisone, prednisone, or triamcinolone within 1 year to 1 week prior to SARS-CoV-2 infection index event. See S6 appendix.

(6) Prior encounters were defined as the number of prior billing encounters, calculated as unique days and assuming each outpatient and ED visit is 1 day, in a patient's EHR in the 1 year before SARS-CoV-2 infection index event.

(7) RECOVER site quartiles are based on overall RECOVER patient enrollment numbers. Sites are categorized by the following; quartile 1: <140,000 participants, quartile 2: <220,000 participants, quartile 3: <400,000 participants, quartile 4: ≥ 400,000 participants

(8) Pediatric medical complexity algorithm (PMCA version 3.0): complex/chronic is an algorithm to define pediatric chronic disease and medical complexity using patients’ previous EHR diagnosis codes within the 3 years before the SARS-CoV-2 infection index event. The most conservative definition of the algorithm was used (33).

(9) Full model adjusted for age, sex, race, medical insurance type, healthcare utilization, date of COVID-19 infection, comorbidities, substance use disorder, smoking status (adults only), body mass index glucocorticoid use, and RECOVER enrollment site size

(10): No prior indicated models excluding those with a history of autoimmune disease
