# Supplementary figures and images for: Severity of acute SARS-CoV-2 infection and risk of new-onset autoimmune disease: A RECOVER initiative study in nationwide U.S. cohorts
Source: PLoS One. 2025 Jun 4;20(6):e0324513. doi: 10.1371/journal.pone.0324513 (PMC12136303; doi:10.1371/journal.pone.0324513)

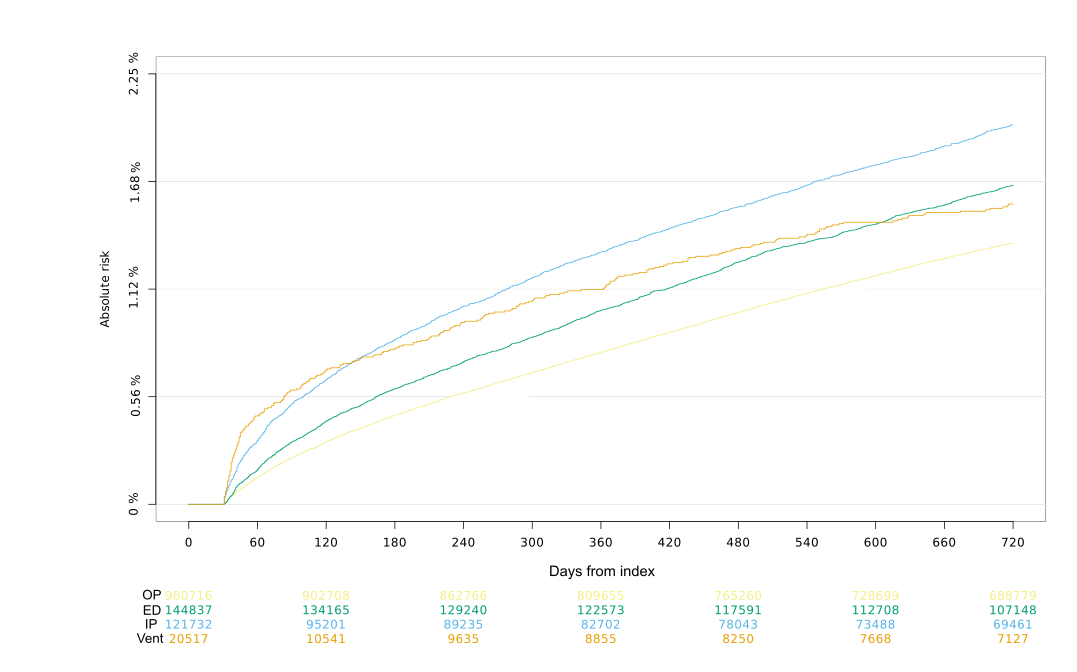

Supplement: S1 Fig — N3C cumulative incidence curve for any incident autoimmune disease with competing risk of death by COVID-19 severity level with 95% CIs. Abbreviations: OP, outpatient; ED, emergency department; IP, inpatient(hospitalized); Vent, hospitalized and on ventilator. (TIFF) [file pone.0324513.s014.tiff]

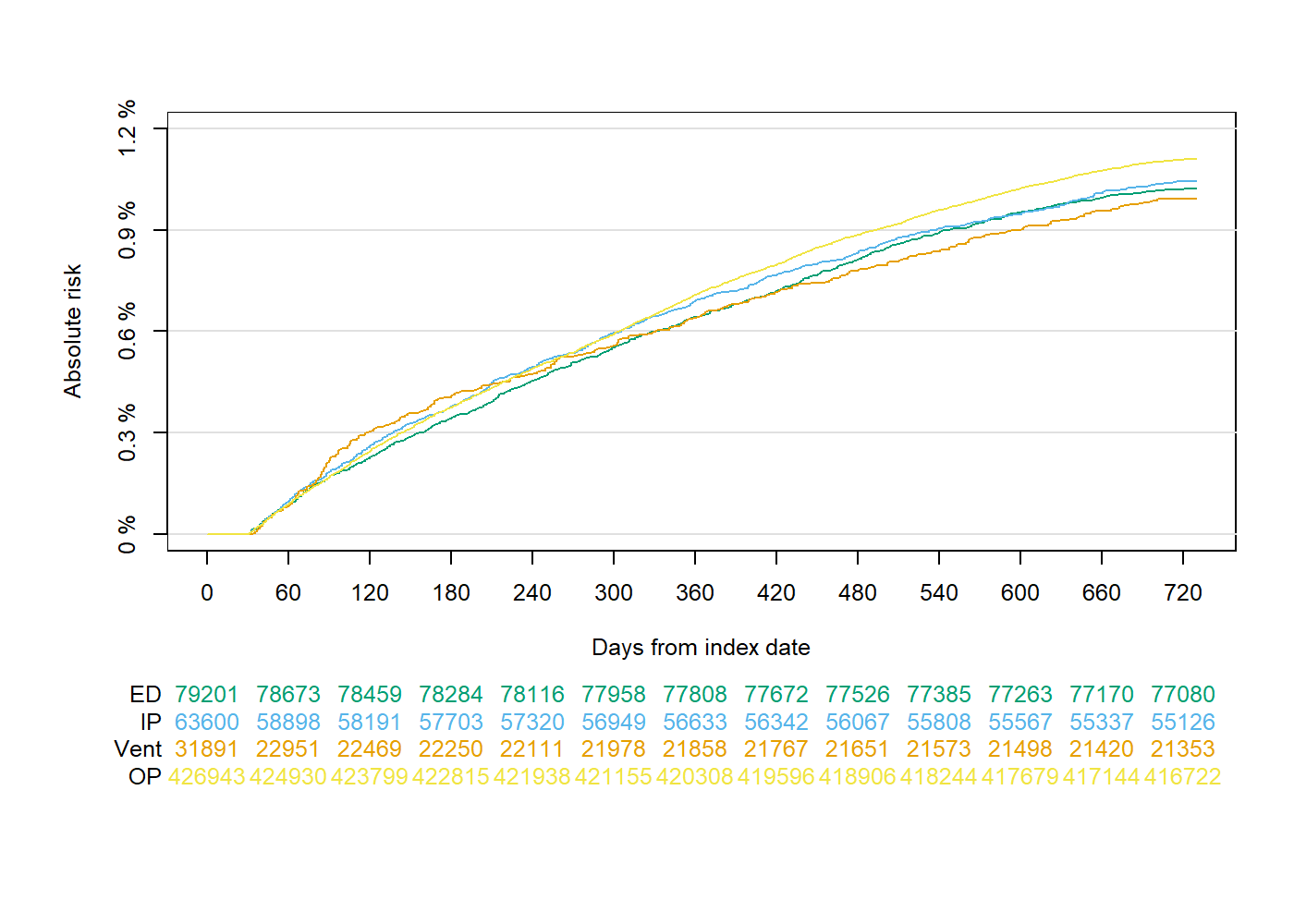

Supplement: S2 Fig — PCORnet cumulative incidence curve for any incident autoimmune disease with competing risk of death by COVID-19 severity level with 95% CIs. Abbreviations: OP, outpatient; ED, emergency department; IP, inpatient(hospitalized); Vent, hospitalized and on ventilator. (TIFF) [file pone.0324513.s015.tiff]

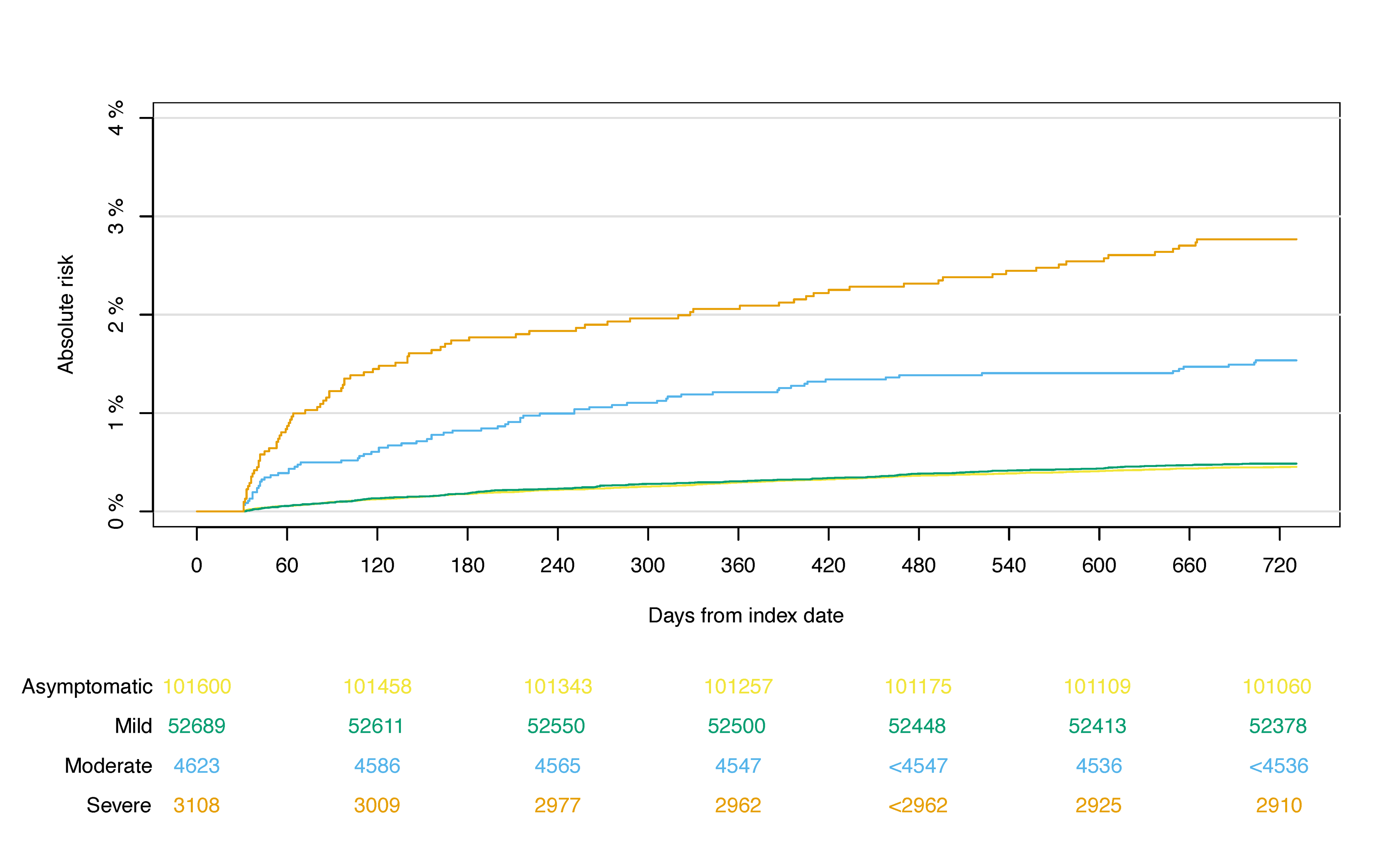

Supplement: S3 Fig — PEDSnet cumulative incidence curve for any incident autoimmune disease with competing risk of death by COVID-19 severity level with 95% CIs. (TIF) [file pone.0324513.s016.tif]

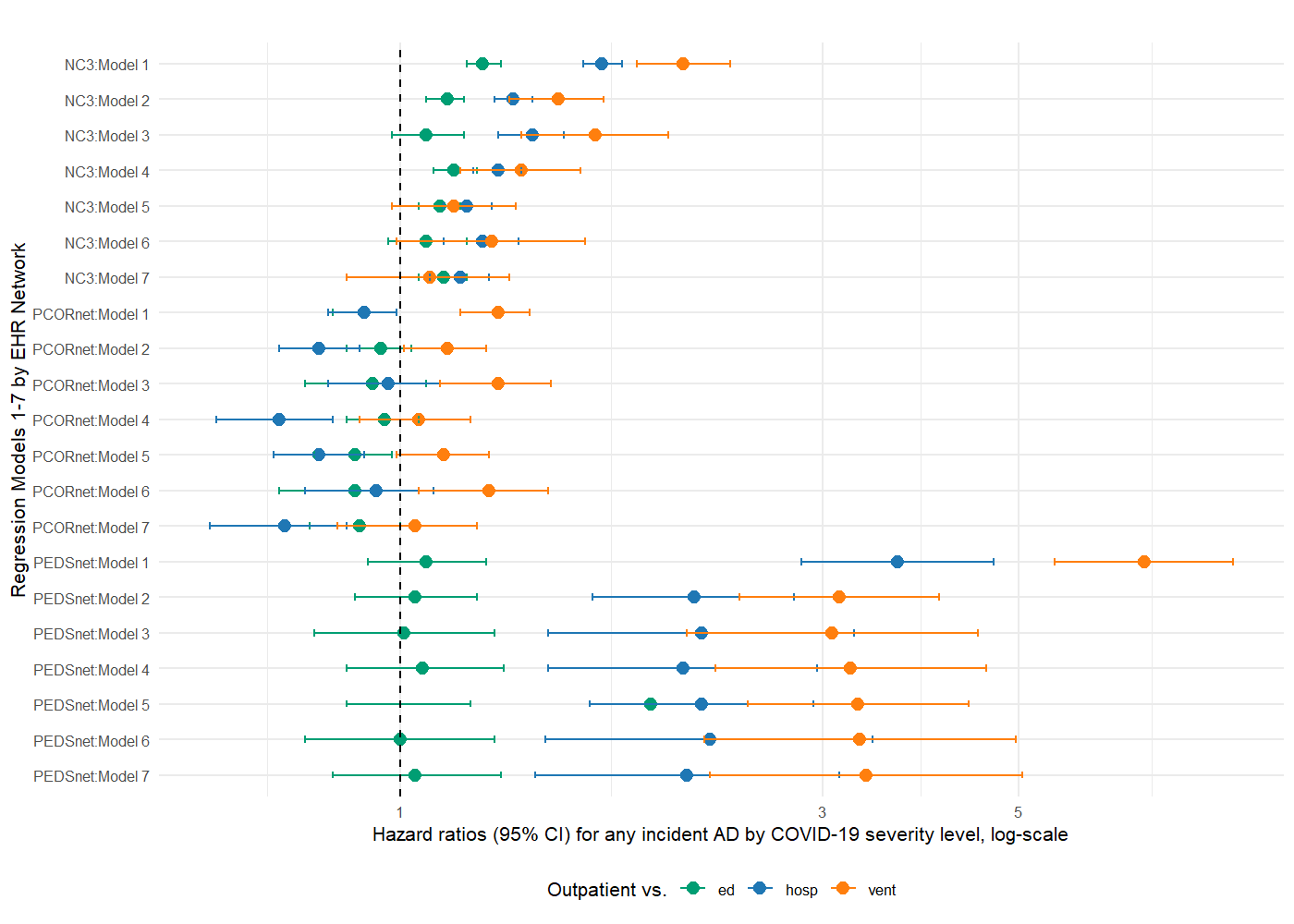

Supplement: S4 Fig — Forest plot of aHRs (95% CIs) for all regression models (1–7) for any incident autoimmune disease by COVID-19 severity levels across EHR networks. (TIFF) [file pone.0324513.s017.tiff]

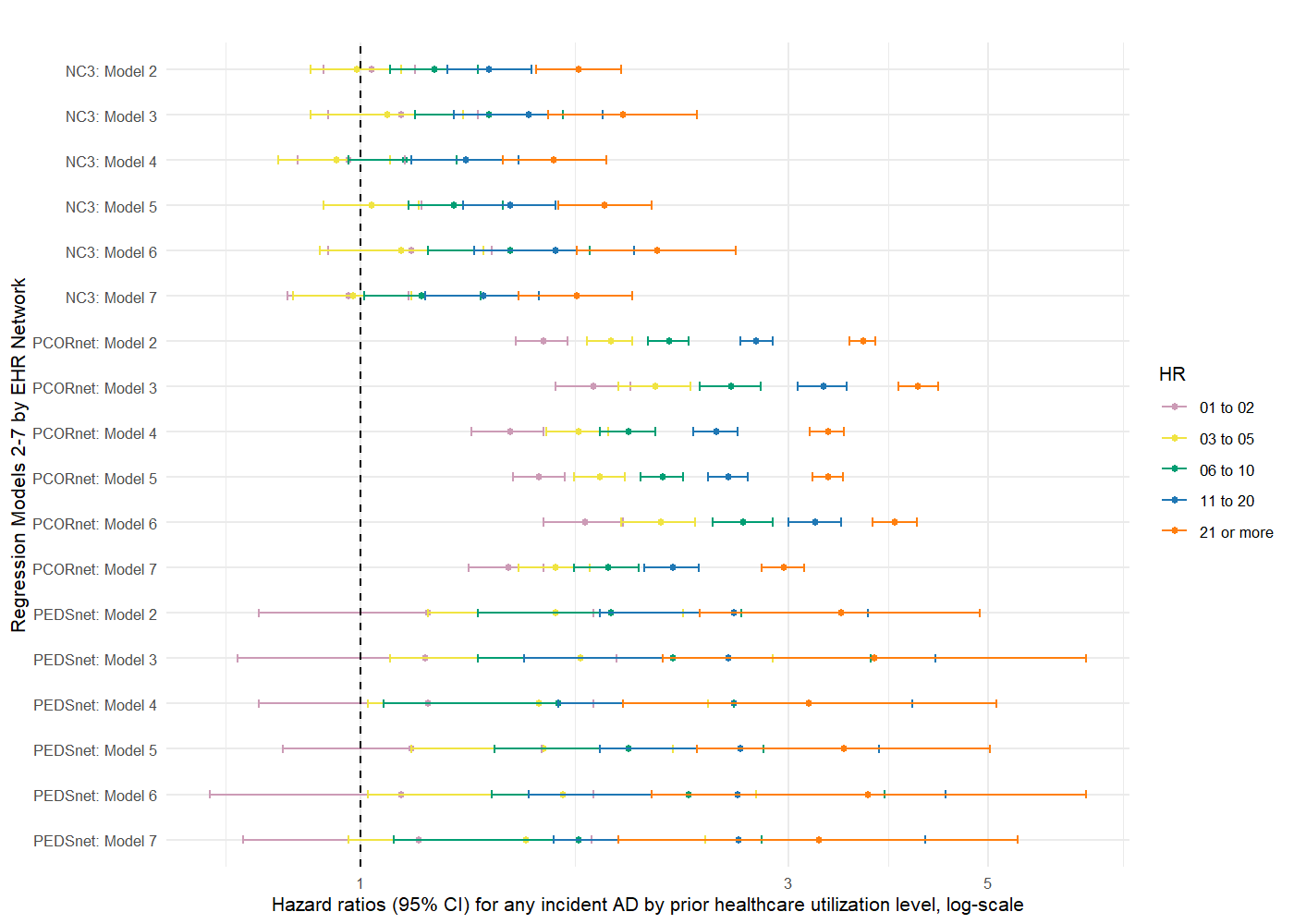

Supplement: S5 Fig — Forest plot of aHRs (95% CIs) for all regression models (1–7) for any incident autoimmune disease by prior healthcare utilization levels across EHR networks. (TIFF) [file pone.0324513.s018.tiff]
