# Supplementary material for: Severity of acute SARS-CoV-2 infection and risk of new-onset autoimmune disease: A RECOVER initiative study in nationwide U.S. cohorts
Source: PLoS One. 2025 Jun 4;20(6):e0324513. doi: 10.1371/journal.pone.0324513 (PMC12136303; doi:10.1371/journal.pone.0324513)
Supplement: Attachment — Submitted filename: Response to Reviewers.docx [file pone.0324513.s020.docx]

We thank the reviewers for their thoughtful comments and suggestions. We have made many revisions and edits to our manuscript in response. All revisions associated with reviewer comments are highlighted in the tracked document.

**Reviewer #1:** The study "Severity of Acute SARS-CoV-2 Infection and Risk of New-Onset Autoimmune Disease" investigates how COVID-19 severity influences the risk of developing autoimmune diseases. The results indicated that severe COVID-19 cases had a significantly higher risk of new-onset autoimmune disease. In adults, diseases like inflammatory arthritis and Sjögren’s syndrome were common, while in children, conditions such as type 1 diabetes and hematological autoimmune diseases were observed. The highest severity category, "hospitalized with ventilation," was associated with a particularly elevated risk, underscoring a dose-response relationship between COVID-19 severity and autoimmune disease development.

Overall, this article well designed and written. I only have some MINOR comments:

1. Introduction Background. The introduction provides a solid foundation by discussing the potential link between viral infections and autoimmunity. To enhance this section, a brief overview of existing literature on COVID-19's role in triggering autoimmune responses would contextualize the study's significance. This addition would underscore the relevance of investigating the association between COVID-19 severity and subsequent autoimmune disease onset.

**Response:** We have expanded our introduction to the manuscript, adding a few sentences and references about the association COVID-19 and the potential for induction of autoimmune disease and the relevance of investigating COVID-19 severity and risk of subsequent autoimmune disease.

“Given these recent reports and the strong biologic possibility that more severe SARS-CoV-2 infection, with higher circulating concentrations of inflammatory cytokines (e.g., interleukin-6) and cytokine storm syndromes in some, may be more strongly linked to triggering of new autoimmune disease, we aimed to investigate the association between SARS-CoV-2 and incident autoimmune diseases.”

2. Justification of Disease Classification. The study defines new-onset autoimmune disease based on the presence of two new ICD codes within 30 days. While this criterion is practical, it may not fully account for transient inflammatory responses that do not culminate in chronic autoimmune conditions. Providing a rationale for this definition or acknowledging it as a limitation would strengthen the study's methodological transparency.

**Response:** While past studies of autoimmune disease risk after SARS-CoV-2 infection have employed just one billing code, we opted to use two ICD codes in the same autoimmune disease group on two separate days within 30 days, but starting 30 days after their index SARS-CoV-2 infection, to identify new autoimmune diseases for several reasons. This definition has been used before with good performance characteristics for rheumatoid arthritis and systemic lupus erythematosus, although there are many autoimmune diseases for which ICD code-based definitions have never been established. Using two codes on separate days is meant to exclude the patients seen for “rule out” of specific autoimmune diseases. Using codes separated by >30 days was impractical given the duration of follow-up, and because patients with non-specific signs and symptoms who are not given a clear diagnosis, are nevertheless often asked to return in several months for re-evaluation and might still receive a billing code for the disease at that time. Thus, we opted to use this definition but acknowledge that such billing code definitions are imperfect.

We have added the following sentence to the discussion acknowledging its limitation.

“Although we employed two billing codes on separate days to identify incident autoimmune disease, rather than one as in most past studies, we acknowledge that no validated definition for all autoimmune disease exists or is feasible in such data, and it may have led to some misclassification of disease status. We began follow-up 30 days after the index SARS-CoV-2 infection to reduce the likelihood that infection-related non-specific symptoms of systemic inflammation were miscoded as new autoimmune disease, but this may still have occurred.”

3. Adjustment for Covariates. The multivariable models adjust for several baseline characteristics, which is commendable. However, the discussion could be enriched by exploring whether factors such as body mass index (BMI), socioeconomic status, or ethnicity might confound the observed association between COVID-19 severity and autoimmune risk. Addressing these variables would provide a more comprehensive understanding of potential confounders.
**Response:** We selected our covariates based on univariable analyses to assess whether they were true confounders (associated with the risk of the predictor and with the risk of the outcome) and included other variables were included for face validity (region of the country, size of the enrollment center, medical insurance (proxy for socioeconomic status), time period of SARS-CoV-2 infection). For the reviewer, we include the tables showing univariable analyses of each predictor with infection severity and of each predictor with incident autoimmune disease. A snippet of the tables of the mentioned variables (BMI, insurance (proxy for socioeconomic status), and race/ethnicity) are displayed below. See Tables A and B on pages 7-10 for the full univariate analysis. Note, a vaccination variable is included in the univariate analysis tables in response to question #4.

| **Table A: Univariate analysis for any new incident AD by each covariate across EHR networks** | | | |
| --- | --- | --- | --- |
|  | Odds ratios (95% CIs) for any new incident AD | | |
|  | **N3C** | **PCORnet** | **PEDSnet** |
| **Body mass index percentile** ^4^ |  |  |  |
| Adults: underweight  PEDS: < 5^th^ percentile | 1.23 (1.04-1.46) | 1.14 (0.90-1.45) | 2.95 (2.07-4.10) |
| Adults: normal  PEDS:5^th^ – 84^th^ percentile | Ref | Ref | 2.47(2.09-2.92) |
| Adults: overweight  PEDS:85^th^ – 94^th^ percentile | 0.99 (0.94-1.05) | 0.99 (0.91-1.07) | 2.14 (1.64-2.75) |
| Adults: moderately obese | 1.01 (0.96-1.07) | 0.98 (0.90-1.06) | --- |
| Adults: severely obese | 1.11 (1.04-1.19) | 1.07 (0.96-1.16) |  |
| Adults: morbidly obese  PEDS:> 95^th^ percentile | 1.16 (1.08-1.23) | 1.08 (0.98-1.19) | 1.99 (1.65-2.38) |
| missing/unknown | 0.57 (0.55-0.60) | 0.35 (0.33-0.38) | Ref |
| **Medical insurance** |  |  |  |
| Private | Ref | Ref | Ref |
| Medicare/Medicaid | 1.1 (1.02-1.18) | 1.24 (1.15-1.33) | 1.48 (1.16-1.89) |
| Other | 0.95 (0.76-1.19) | 0.26 (0.19-0.37) | 0.00 (0-0) |
| Missing/Unknown | 0.70 (0.67-0.74) | 0.87 (0.93-0.93) | 0.89 (0.74-1.08) |
| **Race/ethnicity**^2^ |  |  |  |
| Non-Hispanic Asian | 0.87 (0.78-0.98) | 0.76 (0.65-0.89) | 0.84 (0.71-0.98) |
| Non-Hispanic Black | 0.93 (0.89-0.98) | 0.91 (0.85-0.98) | 0.69 (0.55-0.86) |
| Non-Hispanic White | Ref | Ref | Ref |
| Non-Hispanic Other/Multiple | 0.73 (0.65-0.82) | 0.88 (0.79-0.97) | 1.22 (0.74-1.89) |
| Hispanic or Latino | 0.88 (0.84-0.93) | 0.86 (0.80-0.93) | 0.84 (0.71-0.98) |
| Missing/unknown | 0.79 (0.73-0.86) | 0.54 (0.49-0.58) | 0.58(0.46-0.73) |

| **Table B: Univariate analysis for severe COVID-19 (hospitalized and on ventilator vs outpatient; asymptomatic vs severe) across EHR networks** | | | |
| --- | --- | --- | --- |
|  | Odds ratios (95% CIs) for severe COVID-19 vs least severe COVID-19 | | |
|  | **N3C** | **PCORnet** | **PEDSnet** |
| **Body mass index percentile** |  |  |  |
| Adults: underweight  PEDS: < 5^th^ percentile | 2.69 (2.42-2.99) | 2.63(2.33-2.98) | 4.89 (4.14-5.75) |
| Adults: normal weight  PEDS:5^th^ – 84^th^ percentile | Ref | Ref | 2.45 (2.23-2.68) |
| Adults: overweight  PEDS:85^th^ – 94^th^ percentile | 1.09 (1.04-1.14) | 1.12(1.06-1.17) | 2.61 (2.28-2.98) |
| Adults: moderately obese | 1.21 (1.22-1.36) | 1.20 (1.14-1.26) | -- |
| Adults: severely obese | 1.29 (1.22-1.36) | 1.21 (1.14-1.29) |  |
| Adults: morbidly obese  PEDS:> 95^th^ percentile | 1.68 (1.6-1.77) | 1.45(1.37-1.54) | 2.39 (2.16-2.63) |
| missing/unknown | 0.24 (0.23-0.26) | 1.31 (1.26-1.37) | Ref |
| **Medical insurance** |  |  |  |
| Private | Ref | Ref | Ref |
| Medicare/Medicaid | 1.1 (1.02-1.18) | 1.24 (1.15-1.33) | 1.48 (1.16-1.89) |
| Other | 0.95 (0.76-1.19) | 0.26 (0.19-0.37) | 0.00 (0-0) |
| Missing/Unknown | 0.70 (0.67-0.74) | 0.87 (0.93-0.93) | 0.89 (0.74-1.08) |
| **Race/ethnicity**^2^ |  |  |  |
| Non-Hispanic Asian | 0.87 (0.78-0.98) | 0.76 (0.65-0.89) | 0.84 (0.71-0.98) |
| Non-Hispanic Black | 0.93 (0.89-0.98) | 0.91 (0.85-0.98) | 0.69 (0.55-0.86) |
| Non-Hispanic White | Ref | Ref | Ref |
| Non-Hispanic Other/Multiple | 0.73 (0.65-0.82) | 0.88 (0.79-0.97) | 1.22 (0.74-1.89) |
| Hispanic or Latino | 0.88 (0.84-0.93) | 0.86 (0.80-0.93) | 0.84 (0.71-0.98) |
| Missing/unknown | 0.79 (0.73-0.86) | 0.54 (0.49-0.58) | 0.58(0.46-0.73) |

Additionally, we have added the following text to the Methods section:

“We selected our covariates for our adjusted models based on univariable analyses to assess whether they were true confounders (associated with the risk of the predictor and with the risk of the outcome) and also included other variables for face validity (size of the enrollment center, medical insurance, time period of SARS-CoV-2 infection).

and Results section:

“In our univariable analyses (data not shown), we found age, sex, race/ethnicity, BMI, smoking, and comorbidities to be true confounders (associated with risk of infection and risk of autoimmune disease and thus adjusted for them in our models.”

4. Inclusion of Vaccination Status. Considering the role of COVID-19 vaccination in potentially mitigating autoimmune risk, it would be pertinent to mention whether participants' vaccination statuses were considered or excluded. This information would enhance the study's relevance to current public health considerations.

**Response:** We were disappointed that we were not able to include vaccination status as we agree that this would be valuable information and increase the public health relevance of the study. Our vaccination data proved insufficient for a robust analysis of true vaccination rates. Therefore, we strategically selected a study period characterized by minimal vaccination uptake. To evaluate the potential confounding effect of vaccination, we incorporated it into our models to assess if it should be added into the manuscript, using the following definition: "Patient has evidence of at least 1 dose of COVID-19 vaccination, of any kind (mRNA, protein subunit, unknown labeled doses, and J&J), within 14 to 180 days [inclusive] prior to SARS-CoV-2 index date. Categories: Yes; No/unknown ". The low prevalence of prior vaccination in our study population resulted in negligible changes to the fully adjusted model when the vaccination variable was included and therefore is not included in our final analysis. See snippet of Tables A-B for univariate analysis of vaccination, Table C for raw vaccination numbers, and Table D for the outcomes of the regression model including vaccination. Full Table A-B univariates analyses can be found on pages 7-10.

| **Table A: Univariate analysis for any new incident AD by each covariate across EHR networks** | | | |
| --- | --- | --- | --- |
|  | Odds ratios (95% CIs) for any new incident AD | | |
|  | **N3C** | **PCORnet** | **PEDSnet** |
| **Vaccinated**^1^ | 1.49 (1.27-1.76) | 1.63 (1.22-2.18) | 0.00 (0.00-0.05) |

 (1)Vaccination was defined as a patient having evidence of at least 1 dose of any COVID-19 vaccination [mRNA, protein subunit, unknown labeled doses, and J&J] within 14 to 180 days [inclusive] prior to COVID-19 index.

| **Table B: Univariate analysis for severe COVID-19 (hospitalized and on ventilator vs outpatient; asymptomatic vs severe) across EHR networks** | | | |
| --- | --- | --- | --- |
|  | Odds ratios (95% CIs) for severe COVID-19 vs least severe COVID-19 | | |
|  | **N3C** | **PCORnet** | **PEDSnet** |
| **Vaccinated**^1^ | 0.99 (0.82-1.18) | 0.98 (0.81-1.16) | 0.00 (0-0.01) |

 (1)Vaccination was defined as a patient having evidence of at least 1 dose of any COVID-19 vaccination [mRNA, protein subunit, unknown labeled doses, and J&J] within 14 to 180 days [inclusive] prior to COVID-19 index.

**Table C: Raw vaccination counts across EHR networks**

| **N3C** | | | | |
| --- | --- | --- | --- | --- |
|  | Outpatient  N=980,716 | ED  N=144,837 | Hospitalized  N=121,732 | Hospitalized & on ventilator  N=20,517 |
| **Prior vaccination** |  |  |  |  |
| Yes | 5,829 | 564 | 750 | 121 |
| % vaccinated | 0.59% | 0.39% | 0.62% | 0.59% |
| **PCORnet** | | | | |
|  | Outpatient  N=426,943 | ED  N=79,201 | Hospitalized  N=63,600 | Hospitalized & on ventilator  N=31,981 |
| **Prior vaccination** |  |  |  |  |
| Yes | 1852 | 222 | 251 | 135 |
| % vaccinated | 0.43% | 0.28% | 0.39% | 0.42% |
| **PEDSnet** | | | | |
|  | Asymptomatic  N=101,624 | Mild  N=52,703 | Moderate  N=4,626 | Severe  N = 3,118 |
| **Prior vaccination** |  |  |  |  |
| Yes | 28 | Suppressed* | Suppressed* | 0 |
| % vaccinated | <0.03% | Suppressed* | Suppressed* | (0%) |

*Small cell counts (1 to 10) have been suppressed.

**Table D: Adjusted Hazard Ratios (95% CI) for any new incident autoimmune disease for the regression model, including prior vaccination variable (model 8)**

| **N3C** | | | | |
| --- | --- | --- | --- | --- |
|  | Outpatient  N=980,716 | ED  N=144,837 | Hospitalized  N=121,732 | Hospitalized & on ventilator  N=20,517 |
|  | aHRs (95% CIs) | | | |
| **Model 8:** | Ref | 1.13 (1.08-1.19) | 1.33 (1.27-1.40) | 1.47(1.30-1.67) |
| **PCORnet** | | | | |
|  | Outpatient  N=426,943 | ED  N=79,201 | Hospitalized  N=63,600 | Hospitalized & on ventilator  N=31,981 |
|  | aHRs (95% CIs) | | | |
| **Model 8:** | Ref | 0.96 (0.88-1.03) | 0.82 (0.90-1.02) | 1.14 (1.27-0.85) |
| **PEDSnet** | | | | |
|  | Asymptomatic  N=101,624 | Mild  N=52,703 | Moderate  N=4,626 | Severe  N = 3,118 |
|  | aHRs (95% CIs) | | | |
| **Model 8:** | Ref | 1.04 (0.89-1.22) | 2.15 (1.65-2.79) | 3.14 (2.42-4.07) |

Additionally, the following has been added to the discussion: “….and therefore strategically selected an enrollment period a priori so that vaccination use would naturally be limited in the study population. However, we could not fully assess the role of vaccination on AD incidence during 2 years of follow-up. A subsequent check of raw vaccination numbers confirmed that <1% of the population was vaccinated with any COVID-19 vaccine in the 180 to 14 days prior of their index SARS-CoV-2 infection across EHR networks.

5. Discussion. Suggest adding more discussion on literatures and mechanism and cite relevant references. eg. <https://onlinelibrary.wiley.com/doi/pdfdirect/10.1111/1756-185X.14963>; <https://onlinelibrary.wiley.com/doi/epdf/10.1111/1756-185X.14766>

**Response:** Thank you for these suggestions. As above, we strategically selected an enrollment period a priori to widespread use of COVID-19 vaccines so that vaccination use would be limited in the population. Supplementary Table C shows the very percentages of vaccination in our cohorts. Table D shows models adding prior vaccination, where the findings are very similar to the main models. We have expanded the strengths and limitations within discussion section, adding,

**“**Due to our study period, we were unable to assess the complex relationship between vaccination, autoimmune disease, and COVID-19 that future research must address. Specifically, the potential for vaccination to both mitigate (through SARS-CoV-2 prevention) or increase autoimmune disease risk via mechanisms such as molecular mimicry, adjuvants, and bystander activation requires further study”

And

“….as the established link between severe COVID-19 and autoimmune disease may be confounded by factors such as previous immunosuppressant therapy, potential cross-contaminant use of immunosuppression COVID-19 medications (e.g., combination therapies with disease-modifying anti-rheumatic drugs), differential COVID-19 treatment in fear of cross-contaminant drug use, and increased prevalence of comorbidities associated with severe infection (e.g., diabetes, obesity, older age) within the prior autoimmune disease population.”

**Reviewer #2:** This study presents interesting findings, but there are a few concerns that need to be addressed.

1. The term COVID-19 infection is inaccurate. It is recommended to revise it to SARS-CoV-2 infection for precision.

**Response:** We have revised accordingly.

2. Have you considered addressing reinfection in your analysis? Please clarify.

**Response:** We were also disappointed that we were not able to address re-infection but it was too difficult to separate re-infection from initial infection given the data structure. Accurately addressing SARS-CoV-2 reinfection in the RECOVER EHR networks is complex and is currently an ongoing research topic within the RECOVER EHR cohort. This limitation is mentioned in the discussion section (line 578)

3. Regarding the phrase World Health Organization COVID-19 severity category for adults, while it is clear that you are using WHO guidelines, additional details are needed. Specifically, what codes were employed, and how was the time frame defined?

**Response:** We have included the references to the WHO COVID-19 severity categories (https://cdn.who.int/media/docs/default-source/blue-print/covid-19-therapeutic-trial-synopsis.pdf?sfvrsn=44b83344_1&download=true). The codes used in this study are listed in Supplemental Appendices 7-8. Our timeframe, encompassing encounters from 3 days before to 16 days after the index event (Methods, line 194), was informed by clinicians, data informatics, and previous COVID-19 severity research [PMID:35911666; added as a reference to Methods line 194]. We slightly adapted to include the 3 days prior COVID-19 index, per data informatics recommendation, to account for the acute illness observed in our patients, specifically including cases where ventilation codes preceded in-hospital SARS-CoV-2 testing.

4. For the PEDSnet acute COVID-19 illness severity classification system for children, please provide more detailed information regarding its criteria and application.

**Response:** We provided a supplemental table (S1 Table) depicting mild-severe severity concept definitions and updated the PEDSnet severity definition in the text with the following: “Patients were categorized as asymptomatic, mild, moderate, or severe based on patient COVID-19 symptom clusters identified by using clinically cogent sets of SNOMED CT diagnosis codes examined 7 days before to 13 days after the day of SARS-CoV-2 infection positivity (29). Asymptomatic patients met no criterion for severity. For more information on the PEDSnet mild – severe severity concepts, see S1 Table.”

5. Include the follow-up duration for each cohort used in the study.

**Response:** The follow-up duration in each cohort was up to 24 months from index SARS-CoV-2 infection as stated in the text (line 213). The median follow-up time (in days) by COVID-19 severity level is graphically represented in each of the EHR networks Kaplan-Meier curves. Clearer figures have been provided.

6. The resolution of the figures is too low, rendering them uninterpretable and not informative. Kindly provide high-resolution versions of all figures.

**Response**: We have provided higher resolution figures.

7. It is suggested to refer to PMID: 38437702, not only for discussion but also as a reference for expressions such as SARS-CoV-2 infection.

**Response:** Thank you. We have added this reference to an existing sentence in our discussion.

“Our analysis corroborates and extends findings from recent studies that more severe COVID-19 illness might be associated with increased subsequent autoimmune disease risk “.

**Supplemental tables for reviewer questions:**

| **Table A: Univariate analysis for any new incident AD by each covariate across EHR networks** | | | |
| --- | --- | --- | --- |
|  | Odds ratios (95% CIs) for any new incident AD | | |
|  | **N3C** | **PCORnet** | **PEDSnet** |
| **Age** |  |  |  |
| <1 | -- | -- | Ref |
| 1 to 4 | -- | -- | 1.17 (0.78-1.80) |
| 5 to 9 | -- | -- | 1.56(1.06-2.36) |
| 10 to 15 | -- | -- | 2.21 (1.54-3.29) |
| 16 to 20 | -- | -- | 1.99 (1.39-2.95) |
| 21-35 | Ref | Ref | -- |
| 36-45 | 1.31 (1.24-1.38) | 1.35(1.25-1.47) | -- |
| 46-65 | 1.6 (1.53-1.67) | 1.54(1.42-1.47) | -- |
| 65+ | 1.57 (1.73) | 1.54(1.42-1.66) | -- |
| **Male Sex** | 0.59 (0.57-0.61) | 0.54 (0.52-0.57) | 0.81 (0.71-0.92) |
| **Race/ethnicity**^2^ |  |  |  |
| Non-Hispanic Asian | 0.87 (0.78-0.98) | 0.76 (0.65-0.89) | 0.84 (0.71-0.98) |
| Non-Hispanic Black | 0.93 (0.89-0.98) | 0.91 (0.85-0.98) | 0.69 (0.55-0.86) |
| Non-Hispanic White | Ref | Ref | Ref |
| Non-Hispanic Other/Multiple | 0.73 (0.65-0.82) | 0.88 (0.79-0.97) | 1.22 (0.74-1.89) |
| Hispanic or Latino | 0.88 (0.84-0.93) | 0.86 (0.80-0.93) | 0.84 (0.71-0.98) |
| Missing/unknown | 0.79 (0.73-0.86) | 0.54 (0.49-0.58) | 0.58(0.46-0.73) |
| **Pediatric medical complexity algorithm: complex/chronic** ^7^ | | | |
| Neither chronic nor complex | -- | -- | Ref |
| Chronic | -- | -- | 1.78(1.33-2.32) |
| Complex-chronic | -- | -- | 4.23(3.57-4.99) |
| **Charleson Comorbidity Index** | | | |
| 0 | Ref | Ref | -- |
| 1-3 | 1,8 (1.74-1.86) | 2.09 (1.98-2.21) | -- |
| 4+ | 2.33 (2.23-2.44) | 2.54 (2.39-2.71) | -- |
| **Smoking Status** | | | |
| Never | Ref | Ref | -- |
| Ever | 1.35 (1.27-1.42) | 1.09 (1.02-1.16) | -- |
| Missing/Unknown | -- | 0.76 (0.72-0.81) | -- |
| **Prior substance abuse** | 1.35 (1.27-1.43) | 1.66 (1.53-1.80) | 1.61 (1.04-2.37) |
| **Body mass index percentile** ^4^ |  |  |  |
| Adults: underweight  PEDS: < 5^th^ percentile | 1.23 (1.04-1.46) | 1.14 (0.90-1.45) | 2.95 (2.07-4.10) |
| Adults: normal  PEDS:5^th^ – 84^th^ percentile | Ref | Ref | 2.47(2.09-2.92) |
| Adults: overweight  PEDS:85^th^ – 94^th^ percentile | 0.99 (0.94-1.05) | 0.99 (0.91-1.07) | 2.14 (1.64-2.75) |
| Adults: moderately obese | 1.01 (0.96-1.07) | 0.98 (0.90-1.06) | --- |
| Adults: severely obese | 1.11 (1.04-1.19) | 1.07 (0.96-1.16) |  |
| Adults: morbidly obese  PEDS:> 95^th^ percentile | 1.16 (1.08-1.23) | 1.08 (0.98-1.19) | 1.99 (1.65-2.38) |
| missing/unknown | 0.57 (0.55-0.60) | 0.35 (0.33-0.38) | Ref |
| **Prior glucocorticoid use** ^5^ | 2.16 (2.08-2.24) | 2.63 (1.49-2.79) | 3.27 (2.78-3.84) |
| **Medical insurance** |  |  |  |
| Private | Ref | Ref | Ref |
| Medicare/Medicaid | 1.1 (1.02-1.18) | 1.24 (1.15-1.33) | 1.48 (1.16-1.89) |
| Other | 0.95 (0.76-1.19) | 0.26 (0.19-0.37) | 0.00 (0-0) |
| Missing/Unknown | 0.70 (0.67-0.74) | 0.87 (0.93-0.93) | 0.89 (0.74-1.08) |
| **Prior encounters** ^6^ |  |  |  |
| 0 | Ref | Ref | Ref |
| 1 to 2 | 1.04 (0.93-1.16) | 1.90 (1.73-2.09) | 1.36 (0.99-1.89) |
| 3 to 5 | 1.09 (0.97-1.22) | 259 (2.36-2.85) | 1.99 (1.47-2.76) |
| 6 to 10 | 1.46 (1.21-1.63) | 3.35 (3.06-2.68) | 2.37 (1.73-3.29) |
| 11 to 20 | 1.86 (1.67-2.08) | 4.53 (4.16-4.95) | 3.61 (2.64-5.02) |
| 21+ | 2.88 (2.59-3.19) | 6.56 (6.04-7.12) | 7.05 (5.20-9.74) |
| **Index date period** |  |  |  |
| Apr-June 2020 | Ref | Ref | Ref |
| Jul-Sep 2020 | 0.95 (0.89-1.01) | 0.98 (0.91-1.06) | 0.70 (0.55-0.91) |
| Oct-Dec 2020 | 0.82 (0.78-0.86) | 1.04 (0.98-1.11) | 0.64 (0.51-0.82) |
| Jan-Apr 2021 | 0.89 (0.84-0.94) | 1.07 (0.98-1.15) | 0.72 (0.57-0.91) |
| **RECOVER site size** |  |  |  |
| Quartile 1 | Ref | 2.14 (1.46-3.15) | Ref |
| Quartile 2 | 0.98 (0.91-1.07) | 1.86 (1.27-2.73) | 0.89 (0.70- 1.14) |
| Quartile 3 | 0.84 (0.78-0.9) | 1.84 (1.26-2.69) | 1.03 (0.8126-1.3030) |
| Quartile 4 | 0.69 (0.64-0.74) | ref | 0.64 (0.5212-0.8040) |
| **Vaccinated**^1^ | 1.49 (1.27-1.76) | 1.63 (1.22-2.18) | 0.00 (0.00-0.05) |

 (1)Vaccination was defined as a patient having evidence of at least 1 dose of any COVID-19 vaccination [mRNA, protein subunit, unknown labeled doses, and J&J] within 14 to 180 days [inclusive] prior to COVID-19 index.

| **Table B: Univariate analysis for severe COVID-19 (hospitalized and on ventilator vs outpatient; asymptomatic vs severe) across EHR networks** | | | |
| --- | --- | --- | --- |
|  | Odds ratios (95% CIs) for severe COVID-19 vs least severe COVID-19 | | |
|  | **N3C** | **PCORnet** | **PEDSnet** |
| **Age** |  |  |  |
| <1 | -- | -- | Ref |
| 1 to 4 | -- | -- | 0.54 (0.47-0.62) |
| 5 to 9 | -- | -- | 0.35 (0.30-0.40) |
| 10 to 15 | -- | -- | 0.38 (0.33-0.43) |
| 16 to 20 | -- | -- | 0.38 (0.33-0.42) |
| 21-35 | 0.01 (0.01-0.01) | Ref | -- |
| 36-45 | 1.19 (1.12-1.27) | 1.44 (1.37-1.53) | -- |
| 46-65 | 2.72 (2.6-2.86) | 2.22 (2.11-2.33) | -- |
| 65+ | 7.49 (7.15-7.85) | 6.70(6.44-6.97) | -- |
| **Male Sex** | 1.62 (1.57-1.66) | 1.54 (1.51-1.60) | 1.16 (1.08-1.24) |
| **Race/ethnicity** |  |  |  |
| Non-Hispanic Asian | 1.89 (1.74-2.05) | 2.21 (2.08-2.37) | 1.23(0.97-1.54) |
| Non-Hispanic Black | 2.6 (2.51-2.7) | 1.90 (1.84-1.97) | 2.09 (1.90-2.31) |
| Non-Hispanic White | Ref | Ref | Ref |
| Non-Hispanic Other/Multiple | 1.07 (0.97-1.17) | 1.16 (1.09-1.23) | 1.45 (1.10-1.86) |
| Hispanic or Latino | 1.78 (1.71-1.86) | 1.54 (1.48-1.60) | 1.36 (1.24-1.49) |
| Missing/unknown | 1.18 (1.11-1.26) | 2.39 (2.32-2.46) | 0.73 (0.63-0.83) |
| **Pediatric medical complexity algorithm: complex/chronic** | | | |
| Neither chronic nor complex | -- | -- | Ref |
| Chronic | -- | -- | 1.73 (1.44-2.06) |
| Complex-chronic | -- | -- | 12.75 (11.78-13.79) |
| **Charleson Comorbidity Index** | | | |
| 0 | Ref | Ref | -- |
| 1-3 | 5.97 (5.74-6.2) | 4.22(4.10-4.35) | -- |
| 4+ | 29.82 (28.72-30.97) | 11.97 (11.61-12.34) | -- |
| **Smoking Status** | | | |
| Never | Ref | Ref | -- |
| Ever | 2.85 (2.74-2.97) | 1.53 (1.47-1.58) | -- |
| Missing/Unknown | -- | 1.84 (1.79-1.89) | -- |
| **Prior substance abuse** | 4.81 (4.63-5) | 3.34 (3.22-3.48) | -- |
| **Body mass index percentile** |  |  |  |
| Adults: underweight  PEDS: < 5^th^ percentile | 2.69 (2.42-2.99) | 2.63(2.33-2.98) | 4.89 (4.14-5.75) |
| Adults: normal weight  PEDS:5^th^ – 84^th^ percentile | Ref | Ref | 2.45 (2.23-2.68) |
| Adults: overweight  PEDS:85^th^ – 94^th^ percentile | 1.09 (1.04-1.14) | 1.12(1.06-1.17) | 2.61 (2.28-2.98) |
| Adults: moderately obese | 1.21 (1.22-1.36) | 1.20 (1.14-1.26) | -- |
| Adults: severely obese | 1.29 (1.22-1.36) | 1.21 (1.14-1.29) |  |
| Adults: morbidly obese  PEDS:> 95^th^ percentile | 1.68 (1.6-1.77) | 1.45(1.37-1.54) | 2.39 (2.16-2.63) |
| missing/unknown | 0.24 (0.23-0.26) | 1.31 (1.26-1.37) | Ref |
| **Prior glucocorticoid use** | 2.82 (2.73-2.91) | 1.35(1.30-1.40) | 8.76 (8.33-9.21) |
| **Medical insurance** |  |  |  |
| Private | Ref | Ref | -- |
| Medicare/Medicaid | 5.36 (5.07-5.66) | 6.40(6.11-6.71) | 2.12 (1.91-2.37) |
| Other | 2.22(1.85-2.64) | 1.61 (1.41-1.82) | 1.34 (0.60-2.56) |
| Missing/Unknown | 0.81 (0.77-0.84) | 4.26(4.09-4.44) | 0.67 (0.60-0.72) |
| **Prior encounters** |  |  |  |
| 0 | Ref | Ref | Ref |
| 1 to 2 | 0.48 (0.45-0.52) | 0.40(0.39-0.42) | 0.79 (0.69-0.90) |
| 3 to 5 | 0.48 (0.45-0.52) | 0.35(0.34-0.37) | 0.85 (0.74-0.98) |
| 6 to 10 | 0.59 (0.55-0.77) | 0.44 (0.42-0.47) | 0.95 (0.82-1.10) |
| 11 to 20 | 0.72 (0.68-0.77) | 0.62 (0.60-0.65) | 1.56 (1.35-1.80) |
| 21+ | 1.54 (1.46-1.63) | 1.27 (1.23-1.31) | 5.49 (4.84-6.24) |
| **Index date period** |  |  |  |
| Apr-June 2020 | Ref | Ref | Ref |
| Jul-Sep 2020 | 0.54 (0.52-0.57) | 0.34(0.22-0.35) | 0.40 (0.36 -0.46) |
| Oct-Dec 2020 | 0.34 (0.33-0.36) | 0.39 (0/38-0.40) | 0.29 (0.26-0.32) |
| Jan-Apr 2021 | 0.49 (0.47-0.51) | 0.70 (0.69-0.72) | 0.38 (0.34-0.42) |
| **RECOVER site size** |  |  |  |
| Quartile 1 | Ref | 0.39 (0.33-0.46) | Ref |
| Quartile 2 | 0.73 (0.7-0.78) | 0.92(0.79-1.07) | 1.33 (1.16-1.54) |
| Quartile 3 | 0.45 (0.43-0.47) | 0.81 (0.69-0.94) | 1.76 (1.54-2.02) |
| Quartile 4 | 0.27 (0.26-0.28) | Ref | 0.88 (0.77-1.01) |
| **Vaccinated**^1^ | 0.99 (0.82-1.18) | 0.98 (0.81-1.16) | 0.00 (0-0.01) |

 (1)Vaccination was defined as a patient having evidence of at least 1 dose of any COVID-19 vaccination [mRNA, protein subunit, unknown labeled doses, and J&J] within 14 to 180 days [inclusive] prior to COVID-19 index.
